# Effects of temperature on transcriptome and cuticular hydrocarbon expression in ecologically differentiated populations of desert *Drosophila*

**DOI:** 10.1002/ece3.2653

**Published:** 2016-12-20

**Authors:** William J. Etges, Cássia C. de Oliveira, Subhash Rajpurohit, Allen G. Gibbs

**Affiliations:** ^1^ Program in Ecology and Evolutionary BiologyDepartment of Biological SciencesUniversity of ArkansasFayettevilleAR 72701USA; ^2^ School of Life SciencesUniversity of NevadaLas VegasNV 89919USA; ^3^ Present address: Math and Science DivisionLyon CollegeBatesvilleAR72501USA; ^4^ Present address: Department of BiologyUniversity of PennsylvaniaPhiladelphiaPA19104USA

**Keywords:** adaptation, climate change, desert, gene expression, host cactus, temperature

## Abstract

We assessed the effects of temperature differences on gene expression using whole‐transcriptome microarrays and cuticular hydrocarbon variation in populations of cactophilic *Drosophila mojavensis*. Four populations from Baja California and mainland Mexico and Arizona were each reared on two different host cacti, reared to sexual maturity on laboratory media, and adults were exposed for 12 hr to 15, 25, or 35°C. Temperature differences influenced the expression of 3,294 genes, while population differences and host plants affected >2,400 each in adult flies. Enriched, functionally related groups of genes whose expression changed at high temperatures included heat response genes, as well as genes affecting chromatin structure. Gene expression differences between mainland and peninsular populations included genes involved in metabolism of secondary compounds, mitochondrial activity, and tRNA synthases. Flies reared on the ancestral host plant, pitaya agria cactus, showed upregulation of genes involved in metabolism, while flies reared on organ pipe cactus had higher expression of DNA repair and chromatin remodeling genes. Population × environment (G × E) interactions had widespread effects on the transcriptome where population × temperature interactions affected the expression of >5,000 orthologs, and there were >4,000 orthologs that showed temperature × host plant interactions. Adults exposed to 35°C had lower amounts of most cuticular hydrocarbons than those exposed to 15 or 25°C, including abundant unsaturated alkadienes. For insects adapted to different host plants and climatic regimes, our results suggest that temperature shifts associated with climate change have large and significant effects on transcriptomes of genetically differentiated natural populations.

## 1 Introduction

Understanding current responses to increasing temperatures in natural populations can help to predict future responses to climate change (Hoffmann & Blows, 1994; Williams, Elizabeth, & Samantha, 2003) because temperature is the primary environmental factor determining most organisms' distribution and abundance (Cossins & Bowler, 1987). Numerous case studies indicate that thermal resistance is a key factor limiting insect species distributions (Addo‐Bediako, Chown, & Gaston, 2000; Chown, 2001; Chown & Nicolson, 2004) and there is increasing evidence that some ectotherms exist near their upper thermal thresholds (Jones et al., 2009; Kellermann et al., 2012; Mercader & Scriber, 2008; Stillman, 2002). Therefore, upper thermal limits may suggest physiological limits or relatively similar selective pressures (Mitchell & Hoffmann, 2010). Phenotypic plasticity is expected as an initial response to environmental change (Lande, 2009; Scheiner, 1993) that can itself evolve depending on levels of genetic variation in plasticity (Scheiner & Lyman, 1989, 1991). The rate of adaptation to climatic shifts thus requires standing genetic variation in those phenotypes responding to temperature variation (Hoffmann & Willi, 2008) and knowledge of the magnitude of plastic responses (reviewed in Sgrò, Terblanche, & Hoffmann, 2016). Initial steps toward understanding the consequences of climate change include insights into how whole genome transcriptional responses vary in response to temperature and desiccation stress (Matzkin & Markow, 2009; Rajpurohit, Oliveira, Etges, & Gibbs, 2013; Sinclair, Gibbs, & Roberts, 2007; Sørensen, Nielsen, & Loeschcke, 2007; Telonis‐Scott, Sgrò, Hoffmann, & Griffin, 2016).

Warm deserts and the communities that inhabit them are particularly well suited to a molecule‐to‐ecosystem approach to understanding mechanisms of temperature adaptation because these extreme conditions directly impact organism survival and reproduction, which in turn are directly influenced by complex suites of behavioral, physiological, and biochemical traits. The arid lands of the southwestern USA and northwestern Mexico are predicted to become warmer and drier in the future due to climatic shifts (Cook & Seager, 2013). Thus, it is crucial to understand how organisms are likely to respond to increased desertification associated with climate change, and an important first step is to understand how current biological communities function in warm, arid environments.

Although many studies have examined genetic variation, physiology, and ecology of desert organisms, our understanding of genome‐level adaptation to desert regions is in its early stages (Matzkin, 2012; Matzkin & Markow, 2009; Rajpurohit et al., 2013). The first desert organism with a sequenced genome was *Drosophila mojavensis* (Gilbert, 2007), and the genomes of its close relatives, *D. arizonae* (Lohse, Clarke, Ritchie, & Etges, 2015) and *D. buzzatii* (Guillén et al., 2015), are now available for comparative study. These genomes provide powerful models for an integrated understanding of resistance to the harsh temperature conditions of arid lands through analysis of population genomic divergence and whole‐transcriptome responses to extreme desert conditions. Here, we focus on *D. mojavensis*, a member of the well‐studied cactus–yeast–Dro*sophila* model system, with a rich ecological background for genomic studies (Barker & Starmer, 1982; Barker, Starmer, & MacIntyre, 1990) because it has undergone adaptation to different host plants (Etges, 1990, 1993; Etges, de Oliveira, Noor, & Ritchie, 2010), and diverged into a variety of ecologically distinct desert habitats (Etges, Johnson, Duncan, Huckins, & Heed, 1999; Heed, 1982; Heed & Mangan, 1986). Furthermore, geographically isolated populations in Baja California and mainland Mexico and Arizona separated by the Gulf of California are considered incipient species due to the evolution of behavioral differences caused by courtship song and cuticular hydrocarbon differences (Etges & Ahrens, 2001; Etges, Over, de Oliveira, & Ritchie, 2006; Wasserman & Koepfer, 1977). Thus, *D.  mojavensis* is an excellent model for understanding genomic responses and adaptation to desert environments.

In the deserts and arid lands of the Americas, about half of the ca 100 species in the *D. repleta* group carry out their life cycles in fermenting cactus tissues (Heed, 1982; Wasserman, 1992). The three species, *D. mojavensis* and its two closest relatives, *D. arizonae* and *D. navojoa*, form a monophyletic group endemic to northwestern Mexico and the southwestern USA (Ruiz, Heed, & Wasserman, 1990). Across its geographical range, *D. mojavensis* uses several host cacti including pitaya agria cactus, *Stenocereus gummosus*, in Baja California and organ pipe, *S. thurberi*, and cina cactus, *S. alamosensis* in mainland Mexico and Arizona (Etges et al., 1999; Heed & Mangan, 1986). Isolation from mainland *D. arizonae* occurred ca 1.3 mya after tectonic drift of present‐day peninsular Baja California from southwestern Mexico (Gastil, Phillips, & Allison, 1975), and gene exchange between *D. arizonae* and *D. mojavensis* continued until ca 250 kya based on genomic divergence estimates and ages of chromosomal inversions (Lohse et al., 2015). This was about the same time that *D. mojavensis* colonized mainland Mexico from Baja California (Smith, Lohse, Etges, & Ritchie, 2012). Other host cacti are used by *D. mojavensis* in southern California, but in this study we focused on the well‐studied Baja California and mainland Mexico populations.

A previous study of the effects of desiccation exposure on adult *D. mojavensis* showed striking effects of host cactus, age, and population differences on survivorship, water loss, cuticular hydrocarbon variation, and transcriptome variation (Rajpurohit et al., 2013). Taken together, we hypothesized that transcriptomic and cuticular hydrocarbon responses to temperature variation should also vary among populations of *D. mojavensis*, and be influenced by exposure to different host cactus substrates during pre‐adult stages. Therefore, we assessed whole‐transcriptome responses to temperature variation in multiple populations from different biotic regions of the Sonoran Desert reared on different host plants. We assessed responses in cuticular hydrocarbon composition to temperature differences because of their roles in mate choice (Etges & Ahrens, 2001) and presumed barriers to transcuticular water loss (Gibbs & Rajpurohit, 2010).

## 2 Materials and Methods

### 2.1 Population collections and husbandry

We collected *D. mojavensis* from four locations, two on each side of the Gulf of California: San Quintin and Punta Prieta from Baja California, and Punta Onah, Sonora and Organ Pipe National Monument, Arizona (Table 1; see Etges et al., 2010 for mapped locations). Flies were collected directly from cactus rots by aspiration and by sweep‐netting over baits in the field, returned to the laboratory, and cultured on banana food (Brazner & Etges, 1993) in 35‐mL shell vials at room temperature (20–22°C).

**Table 1:** Origins of the four populations of *Drosophila mojavensis* in this study, the biotic regions of the desert they are located in (Etges et al., 1999), and numbers of flies used to establish laboratory populations. All flies were collected over banana baits in nature unless otherwise noted. The last two digits of each stock number indicate year of collection except for OPNM08^a^

Population	Stock number	Latitude longitude	Number of founders
Punta Onah, Sonora (Central Gulf Coast)	PO07	29°5′23.15″N	472^b^
		112°10′15.59″W	
Organ Pipe National Monument, AZ(Arizona Upland)	OPNM08	31°58′4.88″N	20 isofemale lines
		112°46′5.75″W	
San Quintin, Baja California (San Pedro Martir)	SQ08	30°30′41.88″N	372^c^
		115°53′34.51″W	
Punta Prieta, Baja California (Vizcaino)	PP08	28°52′43.48″N	465
		114°7′28.90″W	

a Ten to 20 adults from each isofemale line collected in May 2007 were combined and mass reared for at least six generations in 2008 to form this stock. Lines were provided by S. Castrenzana.

b Includes ca 80 adults aspirated from agria rots.

c Includes 355 adults that emerged from 3 agria rots.

After several generations in the laboratory, thousands of adult flies from each population were introduced into 12,720 cm^3^ Plexiglas^®^ population cages and allowed to mate for 7 to 10 days. For each of the four populations, eggs were collected from the cages and distributed into six 250‐ml milk bottles containing banana food. These cultures were started at medium larval densities to minimize nutritionally caused maternal/environmental effects caused by mass rearing in vials and maintained in an incubator that cycled from 27 to 17°C on a 14‐hr:10‐hr LD photoperiod. All eclosed bottle‐reared adults were separated by sex daily and aged to maturity on laboratory food in vials in the incubator.

Sexually mature adults of each sex were counted into groups of 100 and then introduced together into population‐specific oviposition chambers. Adults were allowed to mate for several days, and fresh agar–cactus oviposition media was used to collect eggs for 10 hr in 5.5‐cm‐wide Petri dishes. Eggs from each chamber were washed in deionized water, 70% ethanol, sterile deionized water, counted into groups of 200, transferred to a 1‐cm^2^ piece of sterilized filter paper, and placed on pieces of fermenting cactus in the same incubator as described above. Fermenting cactus cultures were initiated in plugged 250‐ml bottles with a 5.5‐cm‐diameter piece of filter paper covering 75 g of aquarium gravel. Bottles were autoclaved and cooled, 75 g of either agria or organ pipe tissues were placed in the bottles, and the bottles were autoclaved again for 8 min at low pressure. After cooling to room temperature, each culture was inoculated separately by injection with a hypodermic needle with 0.5 ml of an aqueous mixture of a pectolytic bacterium, *Erwinia cacticida* (Alcorn et al., 1991), and 1.0 ml of an aqueous solution of seven yeast species found in natural cactus rots (Starmer, 1982): *Dipodascus starmeri*,* Candida sonorensis*,* Candida valida*,* Starmera amethionina*,* Pichia cactophila*,* Pichia mexicana*, and *Sporopachydermia cereana*. The inoculant solutions were prepared with sterile water and a full inoculating loop of each species of bacteria or yeasts. After 1–2 days, all unhatched eggs were counted in order to calculate egg to adult viability and development time. All eclosed adults from each replicate culture bottle were counted daily under CO_2_ anesthesia, separated by sex, and kept on banana food in vials. All cactus culture bottles and vials containing adults were housed in an incubator at 25°C with a 14‐hr:10‐hr LD cycle until the experiments began.

### 2.2 Temperature treatments

Groups of 30 male or female adults were placed into separate 35‐ml glass vials containing banana food and exposed to 15, 25, or 35°C for 12 hr. This time period was chosen to reflect half of a diurnal temperature cycle, and the range of temperatures were chosen to approximate average low and high temperatures in nature (Gibbs, Perkins, & Markow, 2003). After exposure, females were flash‐frozen in liquid nitrogen and stored at −80°C prior to RNA extraction. The experiments always started at 8:00 a.m., so flies were always exposed to the day/light cycle in the incubators. Only 7‐day‐old females were used for RNA extraction and microarray analysis because of the large number of samples, but both females and males exposed to the temperature treatments were included in the CHC analysis. A total of 24 treatments (four populations × two cacti × three temperatures) were included with four replications resulting in 96 groups of females for gene expression assays. The experiment began in February 2009, ca 12–18 months after the flies were captured in the field.

### 2.3 Analysis of epicuticular hydrocarbon variation (CHCs)

After exposure to each of the temperature treatments, 8‐day‐old female and male adults were frozen and stored at −20°C until CHC extraction. Total CHCs were extracted by rinsing individual adults in hexane for 20 min in a 300‐μl glass vial insert (Microliter Analytical Supplies, Suwanee, GA), evaporating off all solvent in a 40°C heating block, and then freezing each sample at −20°C. Individual CHC extracts were redissolved in 5 μl of heptane containing a known amount of docosane (C_22_) as an internal standard. Each sample was subjected to capillary gas–liquid chromatography using an automated Shimadzu GC‐17A (Shimadzu Scientific Instruments, Columbia, MD) fitted with a 15‐m (ID = 0.22 mm) Rtx‐5 fused‐silica column (Restek Corporation, Bellefonte, PA). Injector and detector temperatures were set at 290 and 345°C, respectively, with the injector port in split mode (3:1 ratio), and the column was heated from 200 to 345°C at 15°C/min holding at 345°C for 4 min.

CHC amounts were quantified in all flies by quantifying peak integrations using Class VP 4.2 software provided by Shimadzu with C_22_ amounts used as an internal standard and expressed as nanograms/fly (Etges & Ahrens, 2001; Etges & Jackson, 2001; Stennett & Etges, 1997). All CHC data were log_10_‐transformed to improve normality. We sampled five adults for each combination of population, sex, cactus, and temperature, and MANOVA was used to estimate significant sources of variation using the model:

$$\begin{array}{cc} Y_{ijkl}= & \mu +P_{i}+S_{j}+C_{k}+T_{l}+I_{P\times S}+I_{P\times C}+I_{P\times T}+I_{S\times C}+I_{C\times T}+I_{S\times T}+I_{P\times S\times C}\\ & +I_{P\times C\times T}+I_{P\times S\times T}+I_{C\times S\times T}+I_{P\times C\times S\times T}+E_{ijkl} \end{array}$$

where μ is the grand mean, *P*_*i*_ is the effect of population (two Baja California vs. two mainland populations), *S*_*j*_ is the effect sex, *C*_*k*_ is the effect of host cactus, *T*_*l*_ is the effect of temperature, *I*_P×S_ is the interaction between population and sex, *I*_P×C_ is the interaction between population and cactus, I_P×T_ is the interaction between population and temperature, *I*_S×C_ is the interaction between sex and cactus, I_C×T_ is the interaction between cactus and temperature, *I*_P×S×C_ is the interaction between population, sex, and cactus, and *E*_ijkl_ is the error term. Post hoc Tukey's studentized range (HSD) tests were used to reveal how temperature influenced amounts of individual CHC components. Principal components analysis (PCA) was used to quantify orthogonal combinations of correlated CHCs, and ANOVAs were used each to assess overall sources of variation for each principal component. Canonical discriminant function analysis (CDF) revealed which CHCs were responsible for differences due to temperature treatments. We used SAS/IML to calculate the angle between the CHC vectors associated with each host plant (SAS‐Institute 2004a). All other analyses were performed with SAS (SAS‐Institute 2004b).

### 2.4 RNA isolation, cDNA synthesis, and microarray hybridization

RNA was extracted from groups of ~30 female flies using RNeasy mini‐kits (Qiagen, Valencia, California USA) using the same protocols as in Rajpurohit et al. (2013). RNA samples were stored at −80°C, and double‐stranded cDNA was synthesized with Invitrogen Superscript Double‐Stranded cDNA Synthesis kits. A NanoDrop spectrophotometer (NanoDrop Technologies) was used to quantify each cDNA sample and to verify all samples contained ≥100 ng/μl of cDNA, A260/A280 ≥ 1.8, and A260/A230 ≥ 1.8. cDNA samples were labeled with Cy3 using a NimbleGen One Color DNA Labeling kit.

We used custom Roche NimbleGen 12‐plex microarrays containing 14,528 unique transcripts based on the *D. mojavensis* genome (ver 1.3 released on 14/4/2009). Nine probes per transcript were used for a total of 130,705 probes; each 12‐plex design included 135K probes with controls. Better estimates of average gene expression are expected with these probe sets than any other single measure (Reimers, 2010). A NimbleGen Hybridization System (Hybridization System 4; BioMicro Systems, Inc.) was used for cDNA hybridizations, and intensities of each spot were scanned with a GenePix 4000B scanner (Molecular Devices) and GenePix Pro software at 532 nm (Cy3). Spot intensities were quantile‐normalized (Bolstad, Irizarry, Astrand, & Speed, 2003) with NimbleScan v2.5 software. Gene call files were created using the Robust Multichip Average (RMA) algorithm (Irizarry et al., 2003) that yields approximate normal distributions of fluor intensities across arrays and treatments with our hybridization protocols (Etges et al., 2015).

### 2.5 Statistical analysis

Our experimental design consisted of 96 hybridizations that were analyzed with a mixed‐model ANOVA, PROC MIXED (SAS‐Institute, 2004b):

$$\begin{array}{cc} Y_{ijk}= & \mu +R_{i}+P_{j}+T_{k}+C_{l}++I_{R\times C}+I_{R\times T}+I_{P\times C}+I_{P\times T}+I_{T\times C}+I_{R\times T\times C}\\ & +I_{P\times T\times C}+E_{ijkl} \end{array}$$

where μ is the grand mean, *R*_*i*_ is the effect of geographic region (Baja California vs. the mainland), *P*_*j*_ is the effect of population nested within regions, *C*_*j*_ is the effect of host cactus, *T*_k_ is the effect of temperature, *I*_R×C_ is the interaction between region and cactus, *I*_R×T_ is the interaction between region and temperature treatment, *I*_P×C_ is the interaction between population and cactus nested within region, and *E*_*ijkl*_ is the error term. To correct for multiple comparisons, we calculated false discovery rates, FDR, for the overall ANOVA data and for all pairwise comparisons between treatments (Benjamini & Hochberg, 1995). All comparisons with FDR < 0.01 were included in our analyses.

### 2.6 Bioinformatic analysis

We used DAVID 6.7 and 6.8 beta (Huang, Sherman, & Lempicki, 2009) to identify functional categories that were enriched as a function of each main experimental variable. We used DAVID's default setting of medium stringency to minimize exclusion of potentially interesting gene ontology (GO) terms. Clusters with enrichment scores <1.3, corresponding to *p* > .05, were excluded from further analyses. We submitted GO terms to GO module (Yang, Li, Lee, & Lussier, 2011) to identify false positives included because of the hierarchical nature of the GO terms. After identifying differentially expressed genes and enriched gene clusters, we used FlyMine (Lyne et al., 2007) to identify orthologous genes in *D. melanogaster*. FlyMine and FlyBase (Attrill et al., 2016) were used to explore gene functions and tissue‐specific expression patterns.

## 3 Results

A total of 7213 adult *D. mojavensis* were reared on both host cacti (*n* = 48 cultures). Nested ANOVAs of both egg to adult development time (DEVT) and viability (Table S1) revealed similar life history patterns to those in previous studies (Etges, 1990; Etges et al., 2010). The Arizona population showed significantly longer DEVT than the others (LSMEAN $\overset{¯}{x}$ ± 1 *SE* da, 14.53 ± 0.023), but the Punta Onah, Sonora, and Punta Prieta, Baja California populations, showed equivalent DEVT (both 13.84 ± 0.022 da) with the shortest DEVT observed in San Quintin, Baja California flies (13.02 ± 0.022 da). There was a significant Cactus × Population interaction term due to longer DEVT of both mainland populations, as well as the population from Punta Prieta, Baja California, on agria cactus vs. organ pipe cactus (Figure S1). All four populations showed increases in DEVT when reared on agria vs. organ pipe cactus, due most likely to the more rapid tissue breakdown of agria tissues at 25°C—in all previous studies, cultures were grown on a 14‐hr:10‐hr LD photoperiod and 27:17°C temperature cycle, a mean daily temperature of 22.8°C.

Baja California populations expressed higher egg to adult viability than mainland populations, 82.3 vs. 75.1, two‐tailed t‐test, *p* = .016, and there were significant differences between populations; *F* = 3.37, *p* = .0276, and cactus substrates; *F* = 9.23, *p* = .0042, but these were not apparent in the nested model (Table S1). Agria cactus decreased viability in both mainland populations, but both Baja California populations showed increased viability on both cacti (Figure S1) similar to previous studies (Etges et al., 2010).

### 3.1 Epicuticular hydrocarbon variation

MANOVA revealed that every main and interaction effect in the complete model was significant, *p* < .0001 (Table 2) revealing the sensitivity of CHC expression due to population differentiation, gender, rearing substrates, temperature, and their interactions. We did not employ a nested model to simplify analysis because large‐scale regional differences in CHCs have already been documented (Etges & Ahrens, 2001; Etges & Tripodi, 2008). Principal components analysis revealed eight PCs that accounted for 86.1 percent of the variation in the data (Table S2). ANOVA was performed using the same full factorial model to reveal the nature of each of the eight PCs (Table 3). Population, cactus, and sex influenced each of the first three PCs, and temperature influenced six of eight PCs. Each of the eight PCs showed significant model effect interactions with temperature revealing a pervasive influence of these treatment effects on different covarying combinations of CHCs in this study.

**Table 2:** MANOVA results for cuticular hydrocarbon variation for 8 day old adult male and female *Drosophila mojavensis* from Baja California and mainland Mexico reared on agria and organ pipe cacti exposed to 15, 25 and 35°C for 12 hr

Source of Variation	Wilk's λ	*F*	*df*	*p*
Population	0.0086	17.77	96,437.95	<.0001
Cactus	0.2985	10.72	32,146	<.0001
Population × Cactus	0.1222	4.65	96,437.95	<.0001
Sex	0.0587	73.14	32,146	<.0001
Population × Sex	0.0646	6.83	96,437.95	<.0001
Cactus × Sex	0.5427	3.84	32,146	<.0001
Population × Cactus × Sex	0.2408	2.78	96,437.95	<.0001
Temperature	0.1587	6.89	64,292	<.0001
Population × Temperature	0.0814	2.40	192,871.43	<.0001
Cactus × Temperature	0.2527	4.51	64,292	<.0001
Population × Cactus × Temperature	0.0897	2.29	192,871.43	<.0001
Sex × Temperature	0.3691	2.95	64,292	<.0001
Population × Sex × Temperature	0.0863	2.33	192,871.43	<.0001
Cactus × Sex × Temperature	0.3051	3.70	64,292	<.0001
Population × Cactus × Sex × Temperature	0.1459	2.16	160,728.09	<.0001

**Table 3:** ANOVA results for the first eight CHC principal components^a^ for *Drosophila mojavensis* of increasing temperatures from two Baja California and two mainland populations reared on agria and organ pipe cactus. All *p* < .01 are indicated in bold, *n* = 241

Effect		PC 1		PC 2		PC 3		PC 4		PC 5		PC 6	
		*F*	*p*	*F*	*p*	*F*	*p*	*F*	*p*	*F*	*p*	*F*	*p*
Model	*df*	**3.88**	**<.0001**	**26.95**	**<.0001**	**14.35**	**<.0001**	**17.24**	**<.0001**	**2.77**	**<.0001**	4.91	<.0001
Population	3	**4.77**	**.003**	**324.79**	**<.0001**	**28.84**	**<.0001**	**21.49**	**<.0001**	2.42	.068	**12.41**	**<.0001**
Cactus	1	**11.46**	**.001**	**7.43**	**.007**	**48.01**	**<.0001**	**75.28**	**<.0001**	**10.05**	**.002**	0.15	.699
Population*Cactus	3	2.68	.048	**4.51**	**.005**	**12.12**	**<.0001**	**14.30**	**<.0001**	2.81	.041	2.70	.048
Sex	1	**56.76**	**<.0001**	**10.48**	**.001**	**232.16**	**<.0001**	**289.64**	**<.0001**	1.72	.191	**11.63**	**.001**
Population*Sex	3	0.83	.481	**9.12**	**<.0001**	0.98	.401	**6.11**	**.001**	0.83	.478	**16.09**	**<.0001**
Cactus*Sex	1	**14.05**	**.0002**	**24.26**	**<.0001**	**15.88**	**<.0001**	0.02	.895	4.17	.042	4.80	.030
Population*Cactus*Sex	3	0.57	.638	**5.21**	**.002**	**3.00**	**.032**	**15.61**	**<.0001**	**5.58**	**.001**	**4.52**	**.004**
Temperature	2	**6.70**	**.002**	**7.86**	**.001**	**5.37**	**.005**	**6.84**	**.001**	0.06	.800	**4.41**	**0.014**
Temp*Population	6	1.71	.121	**6.35**	**<.0001**	**5.45**	**<.0001**	**5.20**	**<.0001**	1.36	.257	**5.71**	**<.0001**
Temp*Cactus	2	1.36	.258	**4.46**	**.013**	**4.46**	**.013**	**29.12**	**<.0001**	**8.04**	**.005**	**7.88**	**.001**
Temp*Population*Cactus	6	**3.11**	**.006**	1.48	.186	**5.45**	**<.0001**	1.88	.086	1.28	.282	1.14	.342
Temp*Sex	2	0.27	.766	0.83	.439	**5.82**	**.004**	**7.73**	**.001**	0.31	.578	1.45	.239
Temp*Population*Sex	6	1.77	.108	**3.47**	**.003**	**8.18**	**<.0001**	**3.88**	**.001**	0.68	.563	**2.78**	**.013**
Temp*Cactus*Sex	2	0.80	.452	2.55	.081	**12.72**	**<.0001**	**35.59**	**<.0001**	1.25	.266	0.29	.745
Temp*Pop*Cactus*Sex	5	0.82	.536	1.72	.131	0.95	.452	2.22	.055	**4.22**	**.006**	1.69	.140

Effect		PC 7		PC 8	
		*F*	*p*	*F*	*p*
Model	*df*	**1.68**	**.009**	**1.71**	**.007**
Population	3	0.55	.649	**4.27**	**.006**
Cactus	1	4.05	.046	0.01	.926
Population*Cactus	3	1.12	.341	2.44	.066
Sex	1	0.89	.346	0.05	.818
Population*Sex	3	0.87	.460	2.81	.041
Cactus*Sex	1	0.03	.857	1.34	.249
Population*Cactus*Sex	3	0.26	.852	2.1	.102
Temperature	2	2.38	.095	**4.19**	**.017**
Temp*Population	6	2.1	.055	0.27	.949
Temp*Cactus	2	**3.71**	**.027**	2.23	.111
Temp*Population*Cactus	6	1.09	.373	0.36	.904
Temp*Sex	2	2.02	.136	0.86	.424
Temp*Population*Sex	6	0.57	.758	1.55	.164
Temp*Cactus*Sex	2	2.88	.059	0.48	.622
Temp*Pop*Cactus*Sex	5	**4.44**	**.001**	1.58	.167

a Eigenvalues and percent of total variance for each PC are provided in Table S2.

Post hoc multiple comparisons of the effects of temperature on each of the 31 CHC components and total CHCs per fly revealed that almost all CHCs were reduced in abundance at 35°C. A common pattern for many CHCs was for flies exposed to 15 and 25°C, CHC amounts were not significantly different, but greater than amounts observed at 35°C indicating decreased CHC production from 25 to 35°C (Table 4), consistent with some earlier observations (Markow & Toolson, 1990). Further, some CHC amounts at 15°C were slightly greater than at 25°C. Several CHC components including 7‐ and 9‐hentricontene, 7,27‐pentatricontadiene, C36a, C36b, 9,27‐heptatricontadiene, C38, and C40 were higher in abundance at 25 °C, as were total CHCs per fly. As most of these CHCs are monoenes or alkadienes not expected to decrease cuticular permeability (Gibbs, 1998, 2002), it was unexpected to observe decreased abundances of these CHCs at 35°C in just 12 hr of exposure. The most abundant alkane, 2‐methyloctacosane, should be best at increasing desiccation resistance because it is a saturated hydrocarbon, or harder wax, but it also decreased in abundance at 35°C. Thus, there was little or no evidence that responses in observed CHC production at 35°C were associated with altered transcuticular water loss. Of those CHCs previously associated with differences in sexual attractiveness (Etges & Tripodi, 2008), both C_34_ dienes shared this pattern of lowered expression at 35°C, and only one of the C_37_ dienes, 9,27‐heptatricontadiene, showed a significant response to temperature where higher amounts were expressed at 25°C (Table 4). This contrasted with increased total C_37_ abundance (35‐methylhexatricont‐10‐ene, 9,27‐heptatricontadiene, 8,28‐heptatricontadiene, and 14‐, 16‐, and 12‐hexatricontene) at 31 or 34°C in laboratory food‐reared flies (Markow & Toolson, 1990; Reidy, Toolson, & Markow, 1991).

**Table 4:** Post hoc comparisons of means of the 31 epicuticular hydrocarbon components in male and female *Drosophila mojavensis* included in this study in the three experimental temperature treatments: 15, 25, and 35°C. Mean hydrocarbon amounts are expressed as ng/fly. Groupings are from Tukey's studentized range (HSD) tests. *p* values are from ANOVA

Hydrocarbon	ECL^a^	15° vs 25° vs 35°	*p*
2‐methyloctacosane	C_28.65_	32.123, 33.063 > 24.187	<.0001
2‐methyltricontane	C_30.65_	86.199, 92.060, 85.605	ns
7‐ and 9‐hentricontene	C_30.78_	4.789 < 5.655 > 4.133	<.0001
Unknown	C_32_	6.557 > 4.936 < 7.745	ns
Unknown alkene	C_33br1_	0.393, 0.394 > 0.315	ns
11‐and 13‐methyldotricontane	C_33br2_	5.478, 5.253 > 4.214	.018
Unknown alkene	C_33br3_	4.227 > 3.810 > 3.368	.028
31‐methyldotricont‐8‐ene	C_32.47_	31.536, 29.232 > 24.369	.001
31‐methyldotricont‐6‐ene	C_32.56_	2.330, 2.313 > 1.886	.007
8,24‐tritricontadiene	C_32.63_	24.969, 24.644 > 21.526	.047
7,25‐tritricontadiene	C_32.70_	23.910, 21.640 > 17.409	<.0001
10‐, 12‐, and 14‐tritricontene	C_32.79_	10.5087, 11.495 > 8.832	ns
Unknown	C_32.86_	3.619 > 2.959, 2.586	.001
8,26‐tetratricontadiene	C_34diene1_	9.140, 10.323 > 8.300	ns
6,24‐ and 6,26‐tetracontadiene	C_34diene2_	22.293, 24.015 > 17.284	<.0001
10‐, 12‐, and 14‐tetratricontadiene	C_34ene_	9.710, 11.752 > 8.401	.001
33‐methlytetratricont‐10‐ene	C_35alk1_	11.120, 10.422 > 8.257	ns
33‐methlytetratricont‐8‐ene	C_35alk2_	13.977, 12.931, 11.727	ns
Unknown alkene	C_35alk3_	21.825, 20.430, 19.349	ns
9,25‐pentatricontadiene	C_34.59_	71.052, 69.730 > 48.576	<.0001
8,26‐pentatricontadiene	C_34.66_	290.40, 272.29 > 231.18	.001
7,27‐pentatricontadiene	C_34.73_	23.750 ≤ 28.535 ≥ 25.468	.014
Unknown diene	C_36a_	13.116 ≤ 14.912 ≥ 11.051	ns
Unknown alkene	C_36b_	5.715 ≤ 7.634 ≥ 6.867	.001
35‐methylhexatricont‐10‐ene	C_37br_	2.493, 2.371, 2.300	ns
9,27‐heptatricontadiene	C_36.5_	18.377 ≤ 20.172 ≥ 15.582	.019
8,28‐heptatricontadiene	C_36.6_	51.625, 59.527, 49.070	ns
14‐, 16‐, and 12‐hexatricontene	C_36.7_	34.320, 38.708, 38.283	ns
Unknown alkene	C_38_	5.306 < 7.510 > 4.663	<.0001
Unknown alkene	C_39_	5.299, 5.307 ≥ 3.793	.007
Unknown alkene	C_40_	3.767 < 4.605 > 3.893	.023
Total hydrocarbons per fly		829.55 ≤ 879.01 ≥ 720.22	.003

a Equivalent chain length based on relative retention times with known standards.

**Figure 1:**
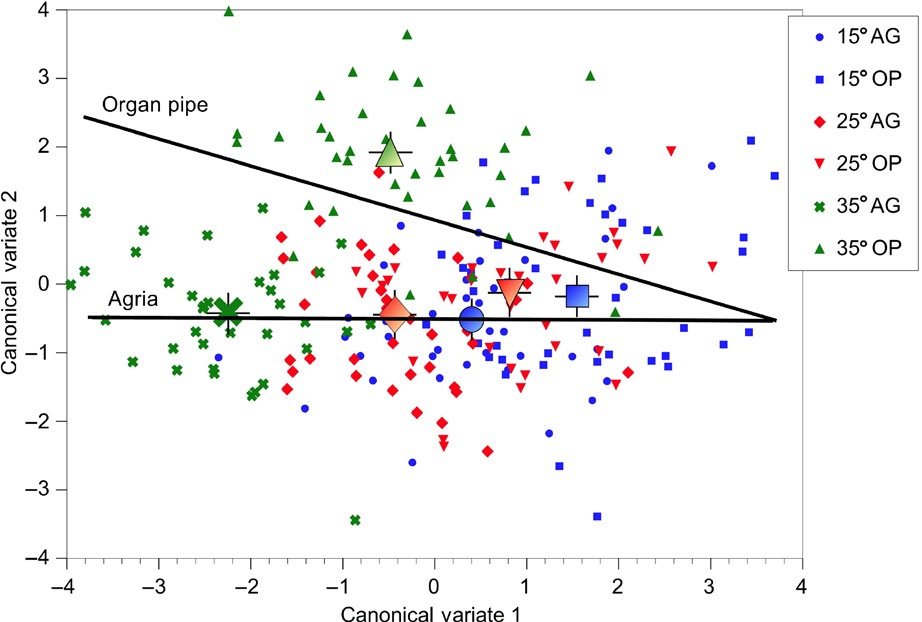
Results of the canonical discriminant function analysis for *Drosophila mojavensis* cuticular hydrocarbons for the first two canonical variates. Differences due to temperature were statistically significant in agria‐reared flies (Wilk's λ = 0.138, *F* = 4.31, *df* = 62/158, *p* < .0001) and organ pipe‐reared flies (Wilk's λ = 0.222, *F* = 2.86, *df* = 62/158, *p* < .0001). The six cactus–temperature class means are plotted showing that much of the variation in CV2 was due to organ pipe‐reared adults exposed to 35°C. The two plotted lines are the regressions for agria vs. organ pipe‐reared adults

Overall temperature effects were further assessed to visualize CHC variation with canonical discriminant function analysis. Variation between temperatures was significant (Wilk's λ lambda = 0.361, *F* = 4.09, *df* = 62/382, *p* < .0001), and all pairwise Euclidean distances between CHCs at the three temperatures were significantly different (all *p* < .001). Those CHCs that varied significantly due to temperature variation (Table 4) showed increased loadings on CV1 and CV2 as expected (Table S3). The angle between the two vectors of log_10_ CHC amounts for each host cactus was just 4.2 degrees suggesting a subtle, but significant effect of cactus on CHC variation. Assessing host cactus effects at each temperature required splitting the data into six groups revealing how much of the variation in CV2 was due to organ pipe‐reared adults at 35°C (Figure 1). All of the temperature–cactus centroids were significantly different from each other (*p* < .001) except for organ pipe‐reared flies at 15 and 25°C. Thus, effects of temperature variation on 8‐day‐old *D. mojavensis* CHCs depended on the host cacti they were reared on from egg to eclosion even though they were held on laboratory food after eclosion.

### 3.2 Patterns of gene expression

Each of the main effects affected the transcription of >1,400 genes, with temperature affecting 3,294 genes. Overall, nearly 80% of all predicted genes were differentially expressed in response to variation in temperature, region, population, host plant, or interactions between these (Table 5). Over 3,000 genes were differentially expressed, mostly as a result of pairwise and three‐way interactions between main effects. Because DAVID is limited to sets of <3,000 genes, we were forced to restrict our analyses to pairwise comparisons for interaction effects where differences in expression were >1.5‐fold in most cases.

**Table 5:** Mixed‐model ANOVA results showing numbers of differentially expressed genes after filtering for FDR *p* < .01 and > 1.5× fold changes for populations of *Drosophila mojavensis* in this study exposed to 15°, 25°, and 35°C. See text for details

Treatments	No. pairwise differences; FDR *p* < .01	No. unique genes	No. genes with Dmel orthologs	No. pairwise differences; 1.5× fold change cut‐off	No. unique genes; 1.5× cut‐off	No. genes with Dmel orthologs
Main effects						
1. Region (R)	1,432	1,432	954	113	114	49
2. Population (P)	4,648	2,433	1,816	745	348	200
3. Temperature (T)	5,390	3,294	2,490	716	484	374
4. Cactus (C)	2,457	2,457	1,816	6	6	5
Interaction effects						
5. R × C	7,195	3,999	2,711	797	466	272
6. R × T	16,302	4,161	3,013	4,464	1,124	789
7. P × C	19,377	5,450	3,272	6,254	2,030	1,289
8. P × T	43,244	5,650	3,942	22,656	2,962	1,961
9. T × C	17,135	4,837	3,536	4,313	1,368	1,030
10. R × T × C	43,221	5,779	4,004	2,2910	3,253	2,139
11. P × T × C	107,404	7,795	5,271	87,333	6,313	4,075
Column totals	267,805	47,287	32,345	150,307	18,468	12,183

### 3.3 Temperature

Temperature affected the expression of 3,294 genes, including 2,490 with some annotation, that is, *D. melanogaster* orthologs. When all such genes were analyzed using DAVID, we identified five clusters of significantly overrepresented gene ontology (GO) terms (Table 6). The first enriched cluster included 93 genes, 39 of which encoded cytochrome P‐450 proteins. The latter group included orthologs of four of the six “Halloween” genes (Rewitz, O'Connor, & Gilbert, 2007) in the biosynthetic pathway for 20‐hydroxyecdysone, that is, *disembodied, shadow, shade*, and *phantom*. All were upregulated at 15 vs. 25 or 35°C (Table S4). Expression of *desat‐2* also decreased as temperature increased, as expected for maintenance of proper membrane fluidity. This may also explain why lower amounts of CHCs were observed at higher temperatures, most of which have double bonds produced by desaturation of hydrocarbon precursors, for example, myristic acid (C14:0) by *desat‐2* in *D. melanogaster* (Yew & Chung, 2015). The gene clustering term “secondary metabolites biosynthesis, transport, and catabolism” was also included in this cluster, indicating temperature‐related shifts in metabolism of host plant compounds. The second cluster was annotated as peptidase inhibitors, with 10 serpin genes of a total of 32 genes. The third and fourth clusters included genes associated with carbohydrate, amino acid, and lipid metabolism. The final cluster was associated with transcription and translation, including many ribosomal proteins and several genes in the His3 and His4 families (Table 6).

**Table 6:** Gene ontology and enrichment for the effects of temperature in this study. All functional clustering was based on genes with FDR *p* < .01 and > 1.5‐fold change for each treatment effect and interaction

Comparison	No. genes (No. Annotated)	GO term	Enrich score
Temperature	3,294 (2,490)	1. Electron carrier activity, heme binding, secondary metabolites biosynthesis, transport, and catabolism	5.3****
		2. Endopeptidase inhibitor activity	4.2***
		3. Vitamin binding	2.2**
		4. Carboxylesterase activity	2.1**
		5. Ribosome, histones, cuticular proteins	1.8*
1. 15° to 25° upregulated	254 (194)	1. Endoplasmic reticulum	1.3
2. 15° to 25° downregulated	640 (473)	1. Endopeptidase inhibitor activity	6.4****
		2. Tetrapyrrole binding	2.4**
		3. Hydrolase activity	1.8*
		4. Peptidase activity	1.6*
3. 25° to 35° upregulated	806 (550)	1. Response to temperature	3.8***
		2. Cation binding	1.4*
4. 25° to 35° downregulated	1045 (667)	1. Ribosome	7.5****
		2. Glycolysis/pentose shunt	1.8*
5. 15° to 35° upregulated	962 (710)	1. Response to temperature	3.5***
		2. Cation binding	2.2**
6. 15° to 35° downregulated	1,686 (1331)	1. Endopeptidase inhibitor activity	6.6****
		2. Tetrapyrrole binding	5.3****
		3. Ribosome biogenesis	1.9*
		4. Amino acid biosynthesis	1.4*
7. 15° down to 25° upregulated to 35°	45 (25)	No enriched groups	
8. 15° up to 25° downregulated to 35°	52 (37)	No enriched groups	
9. 15° up to 25° upregulated to 35°	34 (28)	1. Response to temperature	3.8***
		2. Cation binding	1.4*
10. 15° down to 25° downregulated to 35°	128 (110)	1. Endopeptidase inhibitor activity	4.1***

*p < .05, *^*^ *p* < .01, *^**^ *p* < .001, *^***^ *p* < .0001.

To explore transcriptome changes in more detail, we examined progressive changes in gene expression as temperature increased. A total of 894 genes differed in gene expression from groups of adults reared at 15 to 25°C, where 254 genes increased in expression and 640 genes were downregulated (Table S4). One enriched cluster for genes upregulated from 15 to 25°C including seven genes associated with endoplasmic reticulum indicating increased intracellular protein processing and storage. For downregulated genes, overrepresented GO categories included clusters for serpins, mannosidases, and proteoglycan recognition proteins (Table 6). Members of these genes have been implicated in immune function, suggesting a reduced immune response at 25°C. We also examined the genes with the greatest changes in expression between 15 and 25°C and found that genes for several vitelline membrane proteins increased in expression, while several chorion protein genes decreased in expression (Table S4).

From 25 to 35°C., 1,852 genes differed in expression including two clusters with overrepresented GO categories in the 550 genes upregulated from 25 to 35°C. The first included only six genes, all of them heat‐shock proteins. The second cluster included 83 annotated genes involved with cation binding and genes involved in histone binding and modification (*ADD1, lid, lox*), as well as *DnaJ‐1* (*Hsp40*). *DnaJ‐1* was one of 12 genes associated with gene clusters involving ‘posttranslational modification, protein turnover, chaperones.’ Further inspection of the upregulated genes revealed that the histone deacetylase, *HDAC‐6,* increased in expression from 25 to 35°C, as did a histone acetyltransferase, *mof*, and other genes involved in post‐translational modifications of histones. Eight genes associated with ubiquitin‐mediated proteolysis were also upregulated, as well as four genes in the histone 3 family. Thus, response to higher temperatures included upregulation of heat‐shock proteins as well as histones, suggesting chromatin modification and altered rates of transcription.

There were 1,045 genes downregulated from 25 to 35°C., of which 668 were annotated. Two enriched gene clusters were identified, the first (59 genes) contained 29 cytoplasmic and two mitochondrial ribosome genes, five tRNA synthetases and two elongation–initiation factors, indicating downregulation of protein synthesis. The second cluster (23 genes) contained six genes associated with the pentose‐phosphate shunt pathway. Inspection of the genes not included in these enriched clusters revealed orthologs associated with hexose (e.g., *Gale, Tpi, FASN1*) and general lipid metabolism (*lipid storage droplet‐1, adipokinetic hormone receptor, midway, CDP diglyceride synthetase, phosphatidylinositol synthase*). In addition, there were 12 candidate CHC genes (Gleason, James, Wicker‐Thomas, & Ritchie, 2009) that were downregulated as temperatures increased including stearoyl‐coA 9‐desaturases (*desat‐2*, CG9743, CG15531), a stearoyl‐coA 11‐desaturase (CG9747), a sphingolipid Δ4 desaturase (*ifc*), a Δ5 desaturase (CG17928), and fatty acid elongases (*baldspot*,* bond*, CG2781, CG5326, CG33110, CG6660). Only a Δ6 desaturase, cytochrome b5‐related (Cyt‐b5‐r), was upregulated at 35°C. The gene *bond* is clearly involved in CHC production (J. Yew, pers. comm).

To further explore temperature effects on gene expression, we examined those genes with the greatest overall change in expression. The 25 genes with the greatest increase in expression from 25 to 35°C included seven heat‐shock proteins, including six of the 12 genes with the greatest increase in expression (Table S4). For the top 25 (13 annotated) downregulated genes, GI10103, orthologous to CG10407 in *D. melanogaster*, had the greatest decrease in expression and is involved with hemolymph juvenile hormone binding suggesting higher temperatures increased JH concentrations in adult hemolymph. The gene with the second largest decrease in expression was *desat‐2*, and there were three other lipid metabolism genes included in this list, CG15829, CG18258, and CG6271. CG13084, associated with chorion formation, was also in this group. Together with decreased expression of CG10407, decreases in lipid metabolism and egg production signaled reduced reproductive effort when adults were exposed to 35°C.

We also examined genes that showed significant shifts in expression (*p* < .01, fold change >1.5×) with comparisons involving all three temperature treatments. The comparisons (Table 6) revealed similar patterns to the two temperature comparisons, that is, gene ontology terms including responses to higher temperatures, cation binding, and endopeptidase activity. Thus, the main effects of temperature revealed consistent influences on gene expression as temperature increased from 15 to 25 to 35°C.

### 3.4 Regional differences between mainland Sonora/Arizona and Baja California

While we included just two populations from each region, we found 1,432 genes were differentially expressed in adult female *D. mojavensis* from Sonora/Arizona vs. Baja California populations (Table 5). Approximately equal numbers of genes were upregulated and downregulated in these two regions (Table S6). Cytochrome P‐450 genes and secondary metabolite metabolism were overrepresented in the first enriched gene cluster. A second cluster contained many candidate genes with inferred functions but few known genes, except for five members of the Jonah gene family involved with serine‐type endopeptidase activity. Two other clusters were enriched for tRNA synthetases. Overall, 26 Cyt‐P450 genes in COG_ONTOLOGY categories related to secondary metabolite metabolism were differentially expressed (Table S6). One of these orthologs, Dmoj\GI17558, encodes Cyp28a5 that is part of a gene family known to detoxify cactus alkaloids (Danielson, MacIntyre, & Fogleman, 1997).

A total of 687 genes with greater expression (FDR *p* < .01, >1.5× fold change) in mainland than Baja California flies included five clusters of overrepresented GO categories. The first (29 genes) included 14 Cyt‐P450 genes, while the second reflected differences in transcriptional activity, including aminoacyl‐tRNA synthetases. The other clusters were enriched in endopeptidases, including serpin genes, extracellular recognition proteins including peptidoglycan recognition proteins and peritrophins, and five small heat‐shock proteins from the Hsp20 gene family (Table S6).

A total of 745 genes were expressed at higher levels in flies from Baja California than Sonora and Arizona of which 482 had *D. melanogaster* orthologs (Table 7). DAVID (Huang et al., 2009) revealed three clusters, including one enriched in GO categories related to peptidase activity, including four Jonah family genes. The Jonah genes are most highly expressed in the digestive tract, but other genes in this cluster are expressed in a variety of larval and adult tissues. The second cluster included several electron transport chain genes, as well as *phm* and 15 other Cyt‐P450 genes. Interestingly, these were different P450 genes than those that were upregulated in mainland populations (above) except for genes orthologous to Cyp6a9 and Cyp6a21 that corresponded to more than one Dmoj ID suggesting problems with annotation (Table S6). The third cluster contained 10 peptidase genes, six of which are highly expressed in the digestive tract, while three have their greatest expression in the adult head (Attrill et al., 2016).

**Table 7:** Gene ontology and enrichment for the effects of region, populations, host cacti on gene expression differences in female *Drosophila mojavensis* in this study. All functional clustering was based on genes with FDR *p* < .01 for each treatment effect

Comparison	No. genes (No. annotated)	GO term	Enrich score
1. Region	1432 (954)	1. Electron carrier activity, heme binding, tetrapyrrole binding, P‐450 gene activity	6.8****
		2. Endopeptidase inhibitor activity	2.9**
		3. tRNA processing	1.9*
		4. tRNA aminoacylation	1.7*
Mainland > Baja California	687 (472)	1. Electron carrier activity, heme binding, tetrapyrrole binding, P‐450 gene activity	3.2***
		2. tRNA processing	2.1**
		3. Cofactor binding	1.3*
Baja California > Mainland	745 (482)	1. Endopeptidase inhibitor activity	4.6****
		2. Secondary metabolites biosynthesis, transport, and catabolism, electron carrier activity, heme binding, tetrapyrrole binding	2.8**
		3. Exopeptidase activity	2.0**
2. Population	745 (200)	1. Peptidase, proteolysis	7.3****
		2. Secondary metabolites biosynthesis, transport, and catabolism, electron carrier	2.5**
		3. tRNA processing	3.1***
		4. Endopeptidase inhibitor activity	3.0***
		5. Nonglucose carbohydrate catabolism	2.6**
3. Baja California	153 (83)		
Punta Prieta > San Quintin	72 (39)	1. Sensory perception	2.5**
		2. Muscle protein	1.9*
San Quintin > Punta Prieta	81 (44)	1. Calcium ion binding, EGF‐like	2.1**
		2. P‐450 gene activity, oxidoreductase	1.9*
4. Mainland (Arizona vs. Sonora)^a^	587 (373)		
Organ Pipe NM > Punta Onah	204 (133)	1. Ribosome biogenesis	2.8**
		2. RNA splicing	2.2**
		3. Methyltransferase	2.0**
		4. Hydro‐lyase activity	1.5*
		5. Helicase activity	1.5*
Punta Onah > Organ Pipe NM	383 (240)	1. EGF‐like region	4.2***
		2. Extracellular glycosylation, signal peptide	2.9**
		3. Extracellular matrix	2.8**
		4. Plasma membrane receptor	2.7**
		5. Antimicrobial response	2.7**
5. Host cactus	2457 (1816)		
Agria > Organ pipe	2094 (1524)	1. Peptidase, proteolysis	13.6****
		2. Secondary metabolites biosynthesis, transport, and catabolism, electron carrier activity, heme binding, tetrapyrrole binding	7.2****
		3. ATP, cellular respiration, electron transport	4.6****
		4. Exopeptidase activity	3.7***
		5. Endopeptidase inhibitor activity	3.7***
		6. Cytochrome‐c oxidase activity	3.6***
		7. NADH dehydrogenase activity	3.2***
		8. Extracellular matrix	2.6**
		9. Mitochondrial membrane	2.6**
		10. TCA cycle	2.4**
Organ pipe > Agria	363 (292)	1. DNA repair	9.0****
		2. DNA replication	8.9****
		3. Nucleosome, chromatin assembly	8.5****
		4. ATP binding	5.0****
		5. Mitosis	2.5**

*p < .05, **p < .01, ***p < .001, ****p < .0001.

^a^Organ Pipe National Monument, Arizona and Punta Onah, Sonora, Mexico.

### 3.5 Population differences

For the two populations each from Sonora/Arizona and Baja California, with populations nested within regions in our statistical model, a total of 2,433 genes were differentially expressed in pairwise comparisons between populations. This was reduced to 745 genes with FDR *p* < .01 and >1.5× fold change (Table 5). Not surprisingly, the majority of significant pairwise differences (84%) involved populations from different regions. Within regions, 587 genes differed in expression between the two mainland populations vs. 153 genes (83 annotated) between the two Baja California populations (Table 7). Cluster analyses using DAVID yielded results similar to those for region (Table S7), where the five most significant clusters (of 10) were generally categorized as peptidase activity, secondary metabolism, tRNA processing, peptidase inhibitor activity, and tRNA aminoacylation.

Comparisons of populations within each region, however, revealed significant differences in expression for different up‐ and downregulated gene clusters (Table 7), suggesting that generalizing transcriptional patterns from small numbers of isolated populations vastly underestimated larger regional differences. Within Baja California, the more northerly San Quintin population showed significantly greater expression for EGF‐like and calcium‐binding genes, including two *nimrod* genes, as well as ten P‐450 genes than the population from Punta Prieta in central Baja California. However, Punta Prieta adults showed increased expression for orthologs enriched for sensory perception including four *Obp* genes, *glass*,* prospero* (a DNA‐binding transcription factor involved in nervous system development), and sensory‐related gene, *retinal degeneration A*. Other enriched clusters included muscle‐related calcium‐binding proteins and transcriptional regulation (Table 7).

Greater numbers of genes showed expression differences between the two mainland populations than between the two Baja California populations (Table 7). A total of 587 genes differed in expression, but only 373 were annotated. For the 204 genes significantly overexpressed in the Organ Pipe National Monument, Arizona population vs. the Punta Onah, Sonora population, there were five significantly enriched gene clusters associated with ribosome biogenesis, RNA splicing, methytransferase activity, hydrolyase activity, and ATP‐dependent helicase activity. Thus, increased spliceosome activity, ribosome manufacture, nitrogen metabolism, and ATPase activity suggest Arizona flies have higher rates of mRNA and protein metabolism associated with increased ATP cycling. For genes overexpressed in the Punta Onah, Sonora population, there were 19 enriched gene clusters with scores >1.3 (Table S7) where the top five enriched clusters included functional categories EGF‐like region (as in the San Quintin vs. Punta Prieta comparison), secreted protein modification, extracellular matrix, plasma membrane, and antimicrobial defense response (Table 7). Thus, closer inspection of local population differences in gene expression revealed functional differences not seen in the broader regional comparisons.

### 3.6 Host plant differences

Pre‐adult rearing on different host cacti affected the expression of 2,457 genes of which 1,816 were annotated (FDR *p* < .01), but only six of these pairwise differences exceeded a fold change of 1.5× (Table 5), and all were overexpressed on agria vs. organ pipe cactus. Of these, five were annotated, with Dmoj_GI17776 and Dmoj_GI20850 involved with peptidase activity and Dmoj_GI11137 associated with transmembrane function in the major facilitator superfamily (MFS). This gene family includes single‐polypeptide secondary carriers involved with transporting small solutes in response to chemiosmotic ion gradients (Mitchell et al., 2015). Dmoj_GI21891 and Dmoj_GI13378 were also overexpressed in agria‐reared flies where the former has phospholipase activity and the latter is orthologous to *flightin* associated with adult muscle development in *D. melanogaster* (Table S8). Thus, for genes exceeding 1.5× fold changes, fermenting agria tissues increased expression of a small number of genes associated with ion membrane transport, protein and lipid catabolism, and muscle fiber development in adult *D. mojavensis*.

Because so many genes differed in expression due to cactus rearing substrates at FDR *p* < .01, but did not reach our 1.5× fold change threshold, we also investigated patterns of functional enrichment without this latter filter. Fifteen overrepresented clusters of GO terms were identified from genes there were differentially expressed at FDR *p* < .01 (Table S8), where the first cluster contained 137 peptidase‐related genes, and second included 41 Cyt‐P450s and 43 other genes related to secondary metabolism including *desat‐2*. Six clusters were associated with mitochondrial functions, particularly the electron transport chain. Three clusters were generally associated with extracellular processes, such as cellulose metabolism, chitinase, and extracellular matrix genes.

Striking differences were seen when genes were separated by their up‐ or downregulation on agria vs. organ pipe cactus. A total of 2,094 genes were expressed at higher levels on agria, while only 363 were upregulated on organ pipe cactus. Because genes upregulated on agria dominated the total pool, our clustering analysis was similar to that for all cactus substrate‐affected genes. Eighteen clusters of GO terms were identified, including peptidases, secondary metabolism, and six mitochondrial function clusters (Table 7). In contrast, genes upregulated on organ pipe cactus were dominated by DNA repair (Table S8), replication, and chromatin structure, with five of six clusters relating to DNA functions. Twelve histone genes were upregulated in flies reared on organ pipe cactus. The remaining cluster of 39 ATP and purine nucleoside‐binding proteins included two Hsp70 genes. The significance of these cactus‐related gene expression differences is striking because they were caused by pre‐adult rearing substrates; all RNA samples were extracted from 8‐day‐old adults reared on laboratory media, further evidence of carryover effects of pre‐adult conditions into adulthood (Etges, de Oliveira, Rajpurohit, & Gibbs, 2016).

### 3.7 Interactions between main effects

Clusters of differentially expressed genes identified by DAVID revealed detailed insights into the five pairwise interactions (Table 5) between regional, host plant, and temperature effects. Three‐way interactions resulted in too many genes (> 3000) to be analyzed with DAVID, but for pairwise interaction terms including temperature, we concentrated on cactus and region interaction terms. There were 4,837 genes, 3,536 with some annotation, which differed in expression for Temperature × Host plant interactions resulting in eight enriched clusters (Table S9). The first included 95 proteases, especially Jonah family and trypsin genes. The second included 28 protease inhibitors, especially serpins. The third and fifth were enriched for Cyt‐P450 and heat‐shock genes, respectively. Each of these general clusters was found in one or both separate main effect analyses.

Inspection of functionally enriched gene clusters identified from up and downregulated genes for each significant contrast of cactus and temperature revealed a detailed portrait of temperature effects not seen in the overall analysis of Temperature × Host plant interactions (Table S9). Although these comparisons are not independent, they revealed consistent increases in expression of P‐450, endopeptidase inhibitor, and juvenile hormone‐binding protein gene clusters at low temperatures. At higher temperatures, Hsp20 and Hsp70 heat‐shock proteins, endopeptidase, and peptidoglycan recognition protein gene clusters were overexpressed, the latter involved with immune response and chitin binding (Table S10). A few enriched clusters were observed that were cactus specific such as α amylase‐glycosyl hydrolase expression at 35°C and increased chorion gene expression in 15°C agria‐reared flies.

For genes showing significant Region × Temperature interactions, DAVID recovered eight clusters of overrepresented GO terms. Several mirrored those found for temperature or region alone. The leading cluster represented a variety of metabolic processes, including secondary metabolism that involve metal ion binding, including three metallothionein genes, *SOD3, desat‐2,* and carbohydrate metabolism genes (Table S11). Chromatin remodeling (*ADD1*) and Halloween (*phm, dib*) genes were also present. Other clusters were enriched for proteases, often expressed in the digestive system, and protease inhibitors. A small cluster (enrichment score 2.3) included five of the six heat‐shock genes that were upregulated from 25 to 35°C. Upon closer inspection of all pairwise region–temperature differences, all of these Hsp20 family heat‐shock genes were upregulated at 35°C in populations from both regions (Table S12).

**Figure 2:**
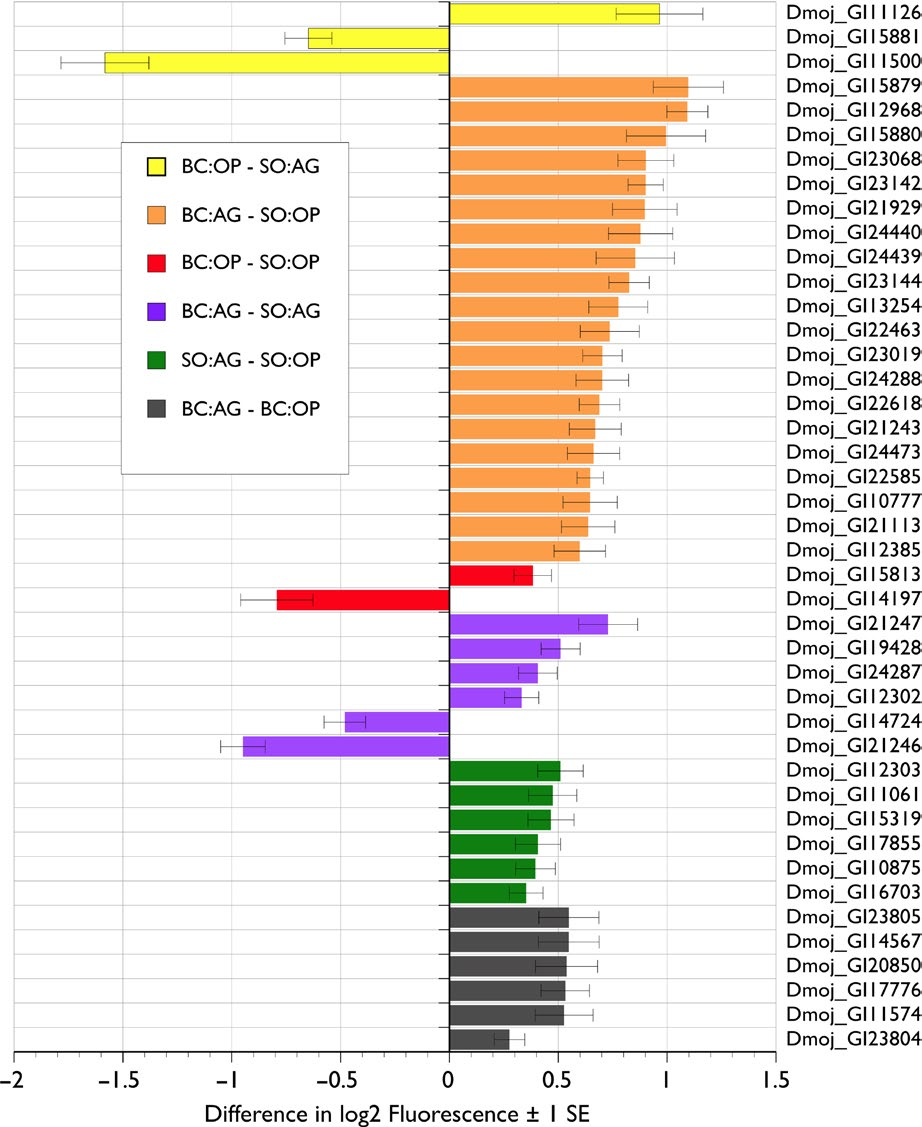
The 43 differentially expressed (FDR *p* < .01, > 1.5 fold ×) neprilysin and protease genes from analyses of Region × Cactus interactions. Most were upregulated in Baja California populations of *Drosophila mojavensis* reared on agria cactus (BC:AG), but a few showed increased expression in mainland populations reared on agria (SO:AG). All were identified in an enriched functional gene cluster by DAVID (Huang et al., 2009) with an enrichment score = 12.6 (*p* < .0001; Table S11)

For Region × Host cactus interactions, 3,999 unique genes showed significant differences in expression (FRD *p* < .01, > 1.5‐fold ×), yet just 2711 were annotated (Tables 5, S13 and S14). The first cluster was enriched for 43 proteolysis/peptidase genes including trypsin encoding genes and the ortholog *Ance*, a member of the peptidase M2 family. There were also members of the M13 class of metalloproteases including neprilysins, involved with peptide hormone metabolism in *Drosophila*, and male and female reproduction including egg laying and sperm use in mated females (Sitnik et al., 2014). Some of these endopeptidase genes are expressed in the female reproductive tracts of *D. mojavensis* and *D. arizonae* (Kelleher & Markow, 2009; Kelleher & Pennington, 2009). Almost all were upregulated in Baja California populations and in flies reared on agria cactus (Figure 2). The second cluster included 47 genes enriched for P‐450 activity and tetrapyrrole binding, and the third cluster was enriched for peptidoglycan function associated with microbial immunity (Tables S13 and S14).

Breaking the Region × Host cactus interactions into pairwise region–cactus comparisons revealed that upregulation of endopeptidases involved agria‐reared flies from both regions (Table S13), and in one case Baja California flies reared on organ pipe cactus. In all cases, mainland populations showed upregulation of GO clusters enriched for secondary metabolites biosynthesis, transport, and catabolism, electron carrier activity, heme binding, or tetrapyrrole binding consistent with overall regional differences (Table 7). A number of other enriched clusters were noted that did not appear in the overall Region × Cactus term analyses were increased glutathione S‐transferase (*Gst*) gene expression in mainland adults reared on both cacti, increased ATP production in the Baja California flies reared on agria vs. mainland flies reared on organ pipe cactus, and increased DNA repair and DNA synthesis in mainland adults reared on organ pipe cactus. *Gst* is involved with catabolism of toxic compounds in insects and shows nucleotide sequence and expression differences between populations of *D. mojavensis* and *D*. *arizonae* (Matzkin, 2008). Thus, the shift to organ pipe cactus in most mainland populations involved increases in secondary compound detoxification afforded by increased *Gst* and P‐450 gene expression with a concordant increase in DNA repair systems.

## 4 Discussion

Temperature is thought to influence a majority of organismal biochemical pathways (MacMillan et al., 2016), and here, brief 12‐hr exposure to ecologically realistic temperature variation directly influenced the expression of approximately a third of the female *D. mojavensis* transcriptome. Together with the number of significant model interactions with temperature, thousands of different genes were up‐ and downregulated that depended on geographic region and host cactus (Table 5) so understanding transcriptomic responses to climate change in natural populations will require knowledge of population history, geographic variation, and local ecology. Although *D. mojavensis* females in our study did not show a full heat‐shock response to high, but nonlethal temperatures as indicated by a large upregulation of *Hsp70* expression, several heat‐shock genes increased in expression from 25 to 35°C. Six of the twelve genes with the greatest expression increase were heat‐shock protein genes, with the second highest being *Hsp70*, and the most significantly overrepresented gene cluster included six *Hsp* genes. Surprisingly, *Hsp70* and *Dnaj‐1* were not included in this cluster, but GI24337, an ortholog of *Droj2*, is heat‐shock protein *DnaJ* included in gene cluster 2. In any case, heat‐shock genes in *D. mojavensis* were upregulated well below lethal temperatures where many heat‐shock proteins act as molecular chaperones to minimize thermal damage to cellular proteins, even though some damage is unavoidable. We also found evidence for increased expression of genes in ubiquitination pathways that would allow *D. mojavensis* to remove damaged proteins more rapidly at high temperatures (Zhou, Campbell, Stone, Mackay, & Anholt, 2012).

In addition to activation of heat‐shock genes, an important part of the classical heat‐shock response is the general downregulation of other genes. Our results also showed that chromatin remodeling affecting gene expression is an important component of the response to temperature. In genes whose expression increased from 25 to 35°C, the second significantly enriched cluster comprised genes associated with chromatin structure and modification. *HDAC‐6S*, for example, upregulates genes associated with responses to abiotic stress (Cho, Griswold, Campbell, & Min, 2005). *lid* is an H3K4 methylase which is also required for H3 acetylation (Lloret‐Llinares, Carré, Vaquero, de Olano, & Azorín, 2008), as is *nej* (Tie et al., 2009). *vig2* tends to increase H3K9me2 levels which are generally associated with a decrease in transcription (Gracheva, Dus, & Elgin, 2009). *mof* and *Axtn9* increase H4 acetylation at the K16 and K9 residues, respectively (Conrad et al., 2012; Mohan et al., 2014). LKRSDH mediates histone arginine methylation caused by 20‐hydroxyecdysone signaling (Cakouros et al., 2008).

Our transcriptome data also indicated changes in energy metabolism associated with temperature. From 25 to 35°C, genes involved in glycolysis and the pentose‐phosphate shunt were downregulated. Decreased expression of *desat‐2* was expected, as this would reduce membrane unsaturation and maintain membrane fluidity. Several other lipid biosynthesis genes were also highly downregulated (Table S4), while several lipases had greater expression. Together, these findings suggest that *D. mojavensis* shifts its fuel source from carbohydrates toward lipids as temperature increases. This shift may be affected by *HDAC‐6*, which downregulates genes associated with glycolysis and biosynthesis (Cho et al., 2005).

The most intriguing difference between flies reared at 15 and 25°C was an increase in vitelline membrane protein expression, while chorion protein expression decreased. The vitelline membrane is laid down before the chorion during oocyte development, so the timing of their expression was different. One potential explanation is that the shift to different temperatures altered the rate of development. Perhaps oocytes matured faster at 25°C so that new oocytes could be initiated in the ovarian stem cell niche. If so, the timing of chorion and vitelline membrane genes could have changed, leading to an apparent difference caused by temperature. Testing this idea would require performing a time series of expression experiments, preferably specific to the ovaries.

The differentially expressed genes and enriched gene clusters due to host cacti overlapped considerably with genes that differed in expression between mainland and peninsular populations (Tables 7 and S8). This may not be surprising, as host plants are largely restricted to either Baja California or the mainland, except for a small population of agria cactus in coastal Sonora, including Punta Onah. Genes encoding mitochondrial proteins were particularly overrepresented, making up seven of the 15 clusters identified by DAVID associated with host cactus effects. Over 5.7 times as many genes (2094/363) were upregulated on agria than on organ pipe cactus. Eight of the resulting 15 clusters enriched for GO terms included mitochondrial function, indicating that flies reared on agria cactus have higher metabolic rates. DAVID also identified six clusters of overrepresented GO terms for genes upregulated on organ pipe cactus. The first included 25 genes associated with DNA repair, and all clusters were associated with aspects of nucleic acid metabolism (Table S8). Thus, flies reared on organ pipe cactus may be subjected to more DNA damage than those reared on agria.

A few broad categories of GO term clusters appeared repeatedly, especially in analyses of interaction effects. For example, clusters containing large numbers of proteases or protease inhibitors were found for two‐way interactions between host plant and region or population (Table S13, Figure 2). The protease clusters often included trypsin orthologs and Jonah family genes some of which have higher expression in the digestive tract, suggesting these gene clusters may reflect populations adapted to feeding on different host plants. Protease inhibitor genes included several members of the serpin family that function as suicide inhibitors of serine proteases and play a wide variety of regulatory roles in development (Reichhart, Gubb, & Leclerc, 2011). Several differentially regulated serpins are involved in immune function, including inhibition of the Toll pathway (Ahmad, Sweeney, Lee, Sweeney, & Gao, 2009; Han, Zhang, Min, Kemler, & Hashimoto, 2000; Tang, Kambris, Lemaitre, & Hashimoto, 2008). Our analysis of genes upregulated in flies reared on agria also found clusters of protease and protease inhibitor genes. This seems counterintuitive, but the substrate specificity of most inhibitors is unknown and it is possible that upregulated proteases and inhibitors act in different pathways. Interactions between temperature and other main effects also uncovered clusters of proteases and protease inhibitors. Several inhibitors are involved in immune responses, and low temperatures improve immune function (Linder, Owers, & Promislow, 2008).

Flies from Baja California and Sonora/Arizona differed in the expression of numerous cytochrome P‐450 genes, many of which are involved in detoxification and metabolism of secondary compounds in their host plants (Fogleman, Danielson, & MacIntyre, 1998; Frank & Fogleman, 1992). Agria and organ pipe cacti contain 25–45% triterpene glycosides and lipids to store carbohydrates and deter herbivory (Kircher, 1977, 1982). When genes upregulated in Sonora/Arizona or Baja California were analyzed separately, 14 different cytochrome P‐450 genes were identified in each comparison, but similar GO term clusters enriched for the COG_ONTOLOGY term ‘secondary metabolite metabolism’ were obtained. Thus, flies from these regions upregulated different sets of detoxification enzymes as responses to their different host plants.

Not all cyt‐P450 proteins are involved in detoxification; four differentially expressed genes are Halloween genes involved in 20‐hydroxyecdysone (20E) biosynthesis. Several ecdysone‐induced genes appeared among genes differentially expressed as a function of population and host plant. Although 20E signaling has been most widely studied for its role in regulating development, it also affects reproduction, behavior, CHC production, and stress resistance in adult *D. melanogaster* (Chiang et al., 2016; Schwedes, Tulsiani, & Carney, 2011). As a steroid hormone, 20E exerts its effects by binding nuclear receptors and changing gene expression patterns: our data revealed that 20E synthesis itself differed among populations and as a function of environmental factors. Decreased expression of some steroid dehydrogenases leads to decreased 20E amounts in early adulthood that causes loss of oenocytes, reduced CHC amounts, and desiccation resistance as well as lowered lifespan in *D. melanogaster* (Chiang et al., 2016). Thus, regulation of 20E biosynthesis in *D. mojavensis* may be a key biochemical pathway that integrates the influences of host plant use, temperature variation, CHC biosynthesis, fitness, and mating success.

### 4.1 Cuticular hydrocarbon expression

Short‐term response to higher temperatures significantly decreased amounts of almost all CHC major components (Table 4) where all experimental model effects were highly significant (Table 2), and the downregulation of 12 CHC candidate desaturases and elongases. Temperature differences resulted in overlapping CHC groups of cactus‐reared flies, with only organ pipe‐reared flies exposed to 35°C as adults displaying altered expression patterns (Figure 1). A cause for the variation in CV2 shown by the CHCs most strongly correlated with it (Table S3) was increased abundance of CHCs at 35°C on organ pipe‐reared flies vs. agria‐reared flies. The latter tended to have the lowest CHCs amounts than any other temperature–host plant combination (results not shown), but organ pipe cactus tended to increase CHC amounts overall consistent with the significant Temperature × cactus interaction in the MANOVA (Table 2). Thus, adult *D. mojavensis* CHCs displayed plastic responses to ecologically realistic temperature differences dependent on the host plant environment experienced in pre‐adult stages.

### 4.2 Climate change, desert ecosystems, and the responsive transcriptome

The Sonoran Desert and adjacent arid lands include well‐studied biotic regions characterized by different plant and animal communities that have been shaped primarily by climatic differences (Shreve & Wiggins, 1964; Turner, Bowers, & Burgess, 1995; Turner & Brown, 1982). From the cooler, Pacific Ocean influenced Baja California coast to the Central Gulf Coast Province on the east side of the peninsula to the Arizona Upland region and Lower Colorado Valley, temperature and precipitation regimes vary considerably in the Sonoran Desert (Hastings, 1964; Hastings & Humphrey, 1969; Hastings & Turner, 1965), and all are within the species range of *D. mojavensis*. Although the species assemblages in these biotic regions are derived from groups in the Miocene (8 mya), the Sonoran Desert developed ca 9 kya, with the current biotic regions evolving 4.5 kya (Betancourt, Van Devender, & Martin, 1990). Baja California separated from the Mexican mainland about 5.5 mya due to tectonic drift and has been in its present position for ca 1 mya (Murphy, 2006; Riddle, Hafner, Alexander, & Jaeger, 2000). Over this time frame, *D. mojavensis* populations expanded throughout these arid lands and now show significant genetic population structure associated with these biotic regions based on variation in chromosomal (Etges et al., 1999) and molecular data (Machado, Matzkin, Reed, & Markow, 2007; Smith et al., 2012).

Interpretations of differences in temperature adaptations between populations of *D. mojavensis* rest on these well‐studied temperature–rainfall differences (Brown & Lowe, 1980). Although global climate models predict that the southwestern desert regions of North America will become warmer and drier in the next few decades (Alwelaie, Hoffman, & Saunders, 1995; Cook, Smerdon, Seager, & Coats, 2014), *D. mojavensis* may be buffered against the immediate impacts of higher temperatures as it has the highest thermal tolerance of any drosophilid studied to date. Larvae and adults survive short‐term exposure up to 44°C (Krebs, 1999; Krebs & Bettencourt, 1999; Stratman & Markow, 1998), and larvae do not display a significant heat‐shock response until 38°C and above (Krebs & Bettencourt, 1999). Mainland adults continue to court, but not mate, up to 38°C (Patton & Krebs, 2001). This is especially relevant to understanding how *D. mojavensis* populations “over‐summer” when temperatures are typically at their annual maxima, cactus rots are few, and fly populations are reduced to low levels (Rockwood‐Sluss, Johnston, & Heed, 1973; R. H. Thomas, pers. comm.). Cactus microclimate temperatures in mainland Sonora and Arizona can exceed 42°C (Gibbs et al., 2003; Hastings & Humphrey, 1969), with average daily July temperatures >31°C in the Central Gulf Coast region of Baja California (Hastings, 1964). Increased expression of small heat‐shock proteins in Sonoran/Arizona adults vs. Baja California flies is consistent with cooler temperatures there because of the influence of Pacific coastal weather patterns. tRNA synthase expression was also higher in Sonora/Arizona populations, suggesting higher overall transcription rates. In flies from Baja California, many electron transport chain and potential digestive proteases were upregulated, suggesting higher rates of metabolic turnover in flies from this region, consistent with findings of Rajpurohit et al. (2013).

## 5 Conclusions

Uncovering genomewide patterns of transcriptional variation is only a first step in marshaling the data needed to predict the consequences of increasing temperature due to climate change in desert ecosystems. Proteomic and metabolomic changes, as well as whole organism fitness effects, will help to uncover the consequences of increasing temperatures to local adaptation and long‐term population persistence. Both up‐ and downregulation of different gene networks influenced by population history and host plant effects emphasizes the need to place temperature‐caused transcriptome variation in a broader ecological and biogeographical context. Expected upregulation of heat‐shock proteins as well as chromatin remodeling genes associated with decreased transcription and reproductive effort showed the pervasive influences of temperature on stress responses, physiology, and fitness. While host plant use, temperature, and desiccation (Rajpurohit et al., 2013) had significant influences on CHC variation, seemingly nonadaptive decreases in CHC amounts and poorly understood temperature effects on sexual selection and isolation at higher temperatures will require more work to understand the multiple roles of CHCs in cuticle permeability and mate choice. Understanding why agria cactus, the ancestral and preferred host, caused increases in expression of protein degradation and energy metabolism genes, including cyt‐P450 genes, and organ pipe cactus increased expression of DNA repair and replication genes (Table 7) will also help to resolve the genetic basis of host plant adaptation in this oligophagic insect.

## Conflict of Interest

None declared.

## Data Archiving

MIAME compliant microarray data are available at Gene Expression Omnibus (http://www.ncbi.nlm.nih.gov/geo/) with accession number GSE72169. All cuticular hydrocarbon data are archived at Dryad doi:10.5061/dryad.r0v81.
