# Supplementary figures and images for: Effects of temperature on transcriptome and cuticular hydrocarbon expression in ecologically differentiated populations of desert Drosophila
Source: Ecol Evol. 2016 Dec 20;7(2):619–37. doi: 10.1002/ece3.2653 (PMC5243788; doi:10.1002/ece3.2653)

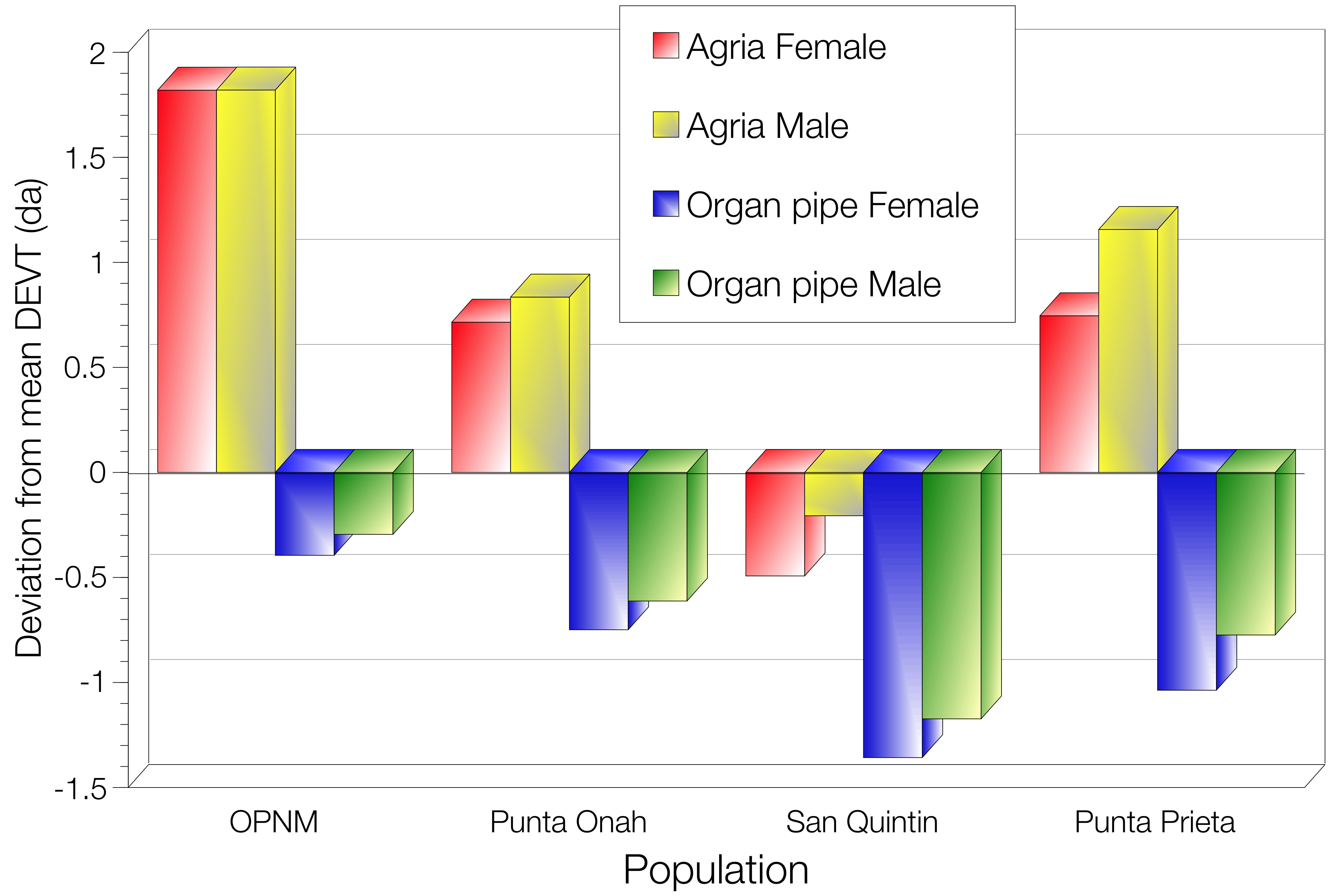


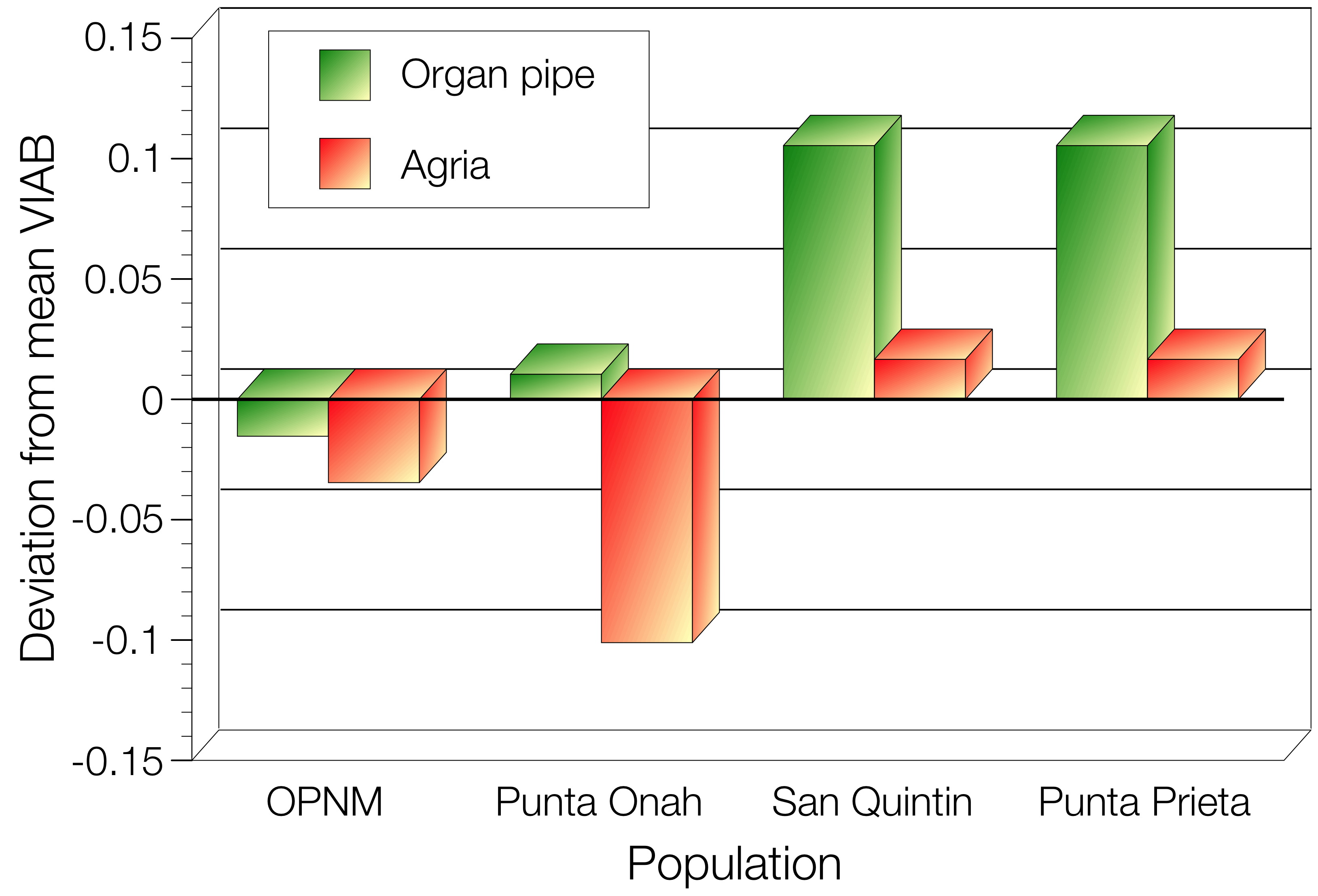

Supplement: Supplementary file 1 [file ECE3-7-619-s001.docx]
