# Supplementary material for: Effects of temperature on transcriptome and cuticular hydrocarbon expression in ecologically differentiated populations of desert Drosophila
Source: Ecol Evol. 2016 Dec 20;7(2):619–37. doi: 10.1002/ece3.2653 (PMC5243788; doi:10.1002/ece3.2653)
Supplement: Supplementary file 2 [file ECE3-7-619-s002.docx]

Supplementary Table 1. Nested ANOVA results for A) egg to adult development time and B) viability for the four populations of *D. mojavensis* cultured on agria and organ pipe cactus substrates in this study at 25 °C. Total n = 7213, total number of cactus cultures = 48**.**

A. log_10_ (Egg to Adult development time)

| Source of Variation | df | Type III SS | Mean Square Ratio ^1^ | F Value | Pr > F |
| --- | --- | --- | --- | --- | --- |
| 1. Region | 1 | 0.0127 | 1/2 | 2.61 | 0.343 |
| 2. Pop (Region) | 2 | 0.0129 | 2/5 | 4.95 | 0.318 |
| 3. Cactus | 1 | 0.0548 | 3/5 | 155.26 | 0.051 |
| 4. Cactus X Region | 1 | 0.0004 | 4/5 | 0.19 | 0.708 |
| 5. Cactus X Pop (Region) ^2^ | 2 | 0.0019 | 5/11 | 3.11 | 0.049 |
| 6. Sex | 1 | 0.0011 | 6/10 | 107.51 | 0.009 |
| 7. Sex X Region | 1 | 0.0001 | 7/10 | 9.23 | 0.093 |
| 8. Sex X Cactus | 1 | 0.0000 | 8/11 | 0.02 | 0.822 |
| 9. Sex X Cactus X Region | 1 | 0.0000 | 9/11 | 0.02 | 0.888 |
| 10. Sex X Pop (Region) | 9 | 0.0000 | 10/11 | 0.02 | 0.983 |
| 11. Error | 82 | 0.0500 |  |  |  |

Supplementary Table 1. cont’d.

B. arcsin (Egg to Adult Viability)

| Source of Variation | df | Type III SS | Mean Square Ratio | F Value | Pr > F |
| --- | --- | --- | --- | --- | --- |
| 1. Region | 1 | 0.1736 | 1/2 | 4.51 | 0.167 |
| 2. Pop (Region) ^2^ | 2 | 0.0770 | 2/5 | 2.37 | 0.297 |
| 3. Cactus | 1 | 0.2287 | 3/5 | 14.09 | 0.064 |
| 4. Cactus X Region | 1 | 0.0129 | 4/5 | 0.79 | 0.468 |
| 5. Cactus X Pop (Region) | 2 | 0.0325 | 5/6 | 0.66 | 0.535 |
| 6. Error | 40 | 0.9905 |  |  |  |

^1^ The mean square ratio used for calculating F ratios. Numbers refer to the listed sources of variation.

^2^ Pop (Region) refers to populations nested within region.
