# Supplementary material for: Effects of temperature on transcriptome and cuticular hydrocarbon expression in ecologically differentiated populations of desert Drosophila
Source: Ecol Evol. 2016 Dec 20;7(2):619–37. doi: 10.1002/ece3.2653 (PMC5243788; doi:10.1002/ece3.2653)
Supplement: Supplementary file 3 [file ECE3-7-619-s003.docx]

Supplementary Table 2. Loadings of each cuticular hydrocarbon on each of the 8 Principal Components in this study.

| Hydrocarbon ^a^ | ECL ^b^ | PC1 | PC2 | PC3 | PC4 | PC5 | PC6 | PC7 | PC8 |
| --- | --- | --- | --- | --- | --- | --- | --- | --- | --- |
| 2-methyloctacosane | C_28.65_ | 0.231 | -0.164 | -0.015 | 0.009 | -0.038 | -0.078 | 0.050 | 0.090 |
| 2-methyltricontane | C_30.65_ | 0.212 | 0.100 | -0.143 | 0.223 | 0.030 | 0.040 | 0.161 | -0.099 |
| 7- and 9-hentricontene | C_30.78_ | 0.216 | 0.190 | 0.114 | -0.085 | -0.084 | -0.157 | 0.005 | 0.209 |
| Unknown | C_32_ | 0.019 | 0.088 | 0.268 | 0.508 | -0.100 | -0.134 | 0.013 | -0.043 |
| Unknown alkene | C_33br1_ | 0.139 | -0.141 | -0.003 | 0.056 | -0.007 | 0.203 | 0.041 | 0.709 |
| 11-and 13-methyldotricontane | C_33br2_ | 0.206 | -0.177 | -0.132 | 0.129 | -0.076 | 0.074 | 0.015 | 0.055 |
| Unknown alkene | C_33br3_ | 0.205 | -0.234 | -0.167 | 0.025 | -0.017 | -0.055 | 0.049 | 0.103 |
| 31-methyldotricont-8-ene | C_32.47_ | 0.228 | -0.074 | -0.161 | 0.150 | -0.025 | -0.062 | 0.020 | -0.018 |
| 31-methyldotricont-6-ene | C_32.56_ | 0.200 | 0.081 | -0.225 | 0.113 | -0.034 | 0.041 | 0.033 | 0.134 |
| 8,24-tritricontadiene | C_32.63_ | 0.237 | -0.078 | -0.071 | 0.046 | -0.054 | -0.072 | 0.062 | -0.011 |
| 7,25-tritricontadiene | C_32.70_ | 0.202 | 0.054 | 0.030 | -0.112 | -0.434 | -0.177 | -0.069 | 0.113 |
| 10-, 12-, and 14-tritricontene | C_32.79_ | 0.197 | -0.016 | 0.103 | -0.110 | 0.363 | -0.257 | 0.091 | 0.087 |
| Unknown | C_32.86_ | 0.073 | -0.179 | 0.246 | 0.242 | 0.550 | -0.125 | -0.015 | 0.053 |
| 8,26-tetratricontadiene | C_34diene1_ | 0.143 | 0.221 | 0.055 | -0.254 | 0.068 | 0.294 | -0.223 | 0.245 |
| 6,24- and 6,26-tetracontadiene | C_34diene2_ | 0.164 | -0.040 | 0.357 | 0.125 | -0.106 | 0.015 | -0.311 | 0.016 |
| 10-, 12-, and 14 tetretricontene | C_34ene_ | 0.124 | 0.008 | 0.344 | 0.027 | -0.011 | 0.143 | -0.491 | -0.163 |
| 33-methlytetratricont-10-ene | C_35alk1_ | 0.199 | -0.133 | -0.162 | 0.068 | 0.068 | 0.287 | -0.356 | -0.138 |
| 33-methlytetratricont-8-ene | C_35alk2_ | 0.211 | -0.034 | -0.209 | 0.072 | 0.118 | 0.166 | -0.244 | -0.210 |
| Unknown alkene | C_35alk3_ | 0.193 | 0.182 | -0.245 | 0.214 | 0.081 | 0.115 | -0.050 | -0.109 |
| 9,25-pentatricontadiene | C_34.59_ | 0.180 | -0.308 | 0.146 | -0.100 | -0.182 | -0.192 | -0.038 | 0.010 |
| 8,26-pentatricontadiene | C_34.66_ | 0.222 | 0.232 | 0.052 | 0.094 | -0.056 | -0.108 | 0.044 | 0.076 |
| 7,27-pentatricontadiene | C_34.73_ | 0.172 | 0.350 | -0.048 | -0.038 | 0.097 | -0.132 | 0.065 | -0.003 |
| Unknown diene | C_36a_ | 0.138 | 0.129 | 0.316 | -0.110 | 0.274 | -0.019 | 0.069 | 0.073 |
| Unknown alkene | C_36b_ | 0.148 | 0.241 | 0.090 | -0.083 | -0.321 | -0.081 | 0.029 | -0.153 |
| 35-methylhexatricont-10-ene | C_37br_ | 0.191 | -0.189 | 0.028 | 0.137 | -0.040 | 0.119 | 0.285 | -0.229 |
| 9,27-heptatricontadiene | C_36.5_ | 0.185 | -0.277 | 0.091 | -0.202 | -0.056 | -0.184 | 0.045 | -0.177 |
| 8,28-heptatricontadiene | C_36.6_ | 0.201 | 0.012 | -0.027 | -0.237 | 0.154 | -0.246 | 0.070 | -0.250 |
| 14-, 16-, and 12-hexatricontene | C_36.7_ | 0.123 | 0.443 | -0.062 | 0.058 | 0.114 | -0.012 | 0.039 | -0.039 |
| Unknown | C_38_ | 0.170 | 0.015 | 0.214 | -0.262 | -0.061 | 0.326 | 0.173 | -0.172 |
| Unknown | C_39_ | 0.161 | -0.045 | 0.034 | -0.309 | 0.122 | 0.379 | 0.278 | -0.057 |
| Unknown | C_40_ | -0.007 | 0.020 | 0.350 | 0.291 | -0.148 | 0.338 | 0.402 | -0.046 |
| Eigenvalue | | 14.515 | 3.400 | 3.255 | 1.788 | 1.148 | 1.023 | 0.836 | 0.715 |
| Percentage of total variance | | 0.468 | 0.110 | 0.105 | 0.058 | 0.037 | 0.033 | 0.027 | 0.023 |

^a^ The 31 epicuticular hydrocarbon components in *D. mojavensis* included - most identified by GCMS; Etges and Jackson (2001), based on all adults in this study reared on both host cacti (n= 224).

^b^ Equivalent chain length based on relative retention times with known standards
