# Supplementary material for: Effects of temperature on transcriptome and cuticular hydrocarbon expression in ecologically differentiated populations of desert Drosophila
Source: Ecol Evol. 2016 Dec 20;7(2):619–37. doi: 10.1002/ece3.2653 (PMC5243788; doi:10.1002/ece3.2653)
Supplement: Supplementary file 4 [file ECE3-7-619-s004.docx]

Supplementary Table 3. Total structure of the first 8 canonical variates (CV) for the 31 cuticular hydrocarbons in this study. CV 1 and 2 loadings in bold correspond to the hydrocarbons that covaried (+/-) and showed significant shifts in amounts due to exposure to 15, 25, or 35 °C. See Table 4.

| Cuticular Hydrocarbon | ECL^1^ | CV 1 | CV 2 | CV 3 | CV 4 | CV 5 | CV 6 | CV 7 | CV 8 |
| --- | --- | --- | --- | --- | --- | --- | --- | --- | --- |
| 2-methyloctacosane | C_28.65_ | **0.356** | **0.224** | 0.075 | -0.190 | -0.136 | 0.089 | 0.175 | 0.062 |
| 2-methyltricontane | C_30.65_ | 0.033 | **0.208** | 0.006 | -0.092 | -0.178 | 0.023 | 0.094 | -0.018 |
| 7- and 9-hentricontene | C_30.78_ | **0.256** | **0.413** | 0.151 | -0.138 | -0.173 | -0.044 | 0.219 | -0.024 |
| Unknown | C_32_ | 0.022 | -0.121 | 0.033 | 0.030 | 0.059 | 0.025 | -0.044 | -0.030 |
| Unknown alkene | C_33br1_ | 0.186 | 0.126 | 0.103 | -0.163 | -0.076 | -0.012 | 0.057 | -0.051 |
| 11-and 13-methyldotricontane | C_33br2_ | 0.155 | 0.123 | 0.040 | -0.103 | -0.138 | 0.014 | 0.044 | -0.055 |
| Unknown alkene | C_33br3_ | 0.157 | -0.011 | 0.072 | -0.136 | -0.126 | 0.136 | 0.025 | -0.035 |
| 31-methyldotricont-8-ene | C_32.47_ | **0.262** | 0.049 | 0.093 | -0.039 | -0.252 | 0.045 | 0.113 | -0.120 |
| 31-methyldotricont-6-ene | C_32.56_ | **0.224** | 0.128 | 0.150 | -0.083 | -0.020 | -0.042 | -0.122 | -0.089 |
| 8,24-tritricontadiene | C_32.63_ | 0.163 | 0.141 | 0.113 | -0.190 | -0.073 | 0.014 | 0.242 | -0.081 |
| 7,25-tritricontadiene | C_32.70_ | **0.286** | **0.353** | 0.230 | -0.067 | -0.172 | -0.027 | 0.122 | 0.103 |
| 10-, 12-, and 14-tritricontene | C_32.79_ | 0.115 | 0.158 | 0.082 | -0.216 | -0.132 | 0.025 | 0.051 | -0.100 |
| Unknown | C_32.86_ | 0.176 | **-0.209** | 0.091 | -0.013 | 0.046 | -0.065 | 0.006 | 0.038 |
| 8,26-tetratricontadiene | C_34diene1_ | 0.162 | **0.301** | 0.058 | -0.044 | -0.010 | 0.098 | 0.251 | 0.054 |
| 6,24- and 6,26-tetracontadiene | C_34diene2_ | **0.288** | **0.306** | 0.169 | -0.101 | -0.029 | 0.122 | 0.080 | -0.051 |
| 10-, 12-, and 14 tetretricontene | C_34ene_ | **0.187** | **0.411** | 0.202 | -0.072 | 0.022 | 0.098 | 0.100 | -0.085 |
| 33-methlytetratricont-10-ene | C_35alk1_ | **0.165** | 0.075 | 0.053 | -0.132 | -0.159 | 0.041 | 0.146 | 0.089 |
| 33-methlytetratricont-8-ene | C_35alk2_ | 0.108 | 0.037 | 0.067 | -0.028 | -0.158 | -0.037 | 0.188 | 0.078 |
| Unknown alkene | C_35alk3_ | 0.085 | 0.017 | 0.084 | 0.023 | -0.146 | -0.003 | 0.118 | -0.011 |
| 9,25-pentatricontadiene | C_34.59_ | **0.251** | 0.147 | 0.137 | -0.175 | -0.092 | 0.114 | 0.093 | 0.089 |
| 8,26-pentatricontadiene | C_34.66_ | **0.262** | **0.288** | 0.015 | -0.042 | -0.183 | 0.002 | 0.112 | -0.024 |
| 7,27-pentatricontadiene | C_34.73_ | 0.043 | **0.362** | 0.032 | -0.074 | -0.189 | -0.049 | 0.192 | 0.091 |
| Unknown alkene | C_36a_ | 0.152 | **0.236** | 0.094 | -0.060 | -0.084 | 0.130 | 0.118 | 0.014 |
| Unknown alkene | C_36b_ | 0.018 | **0.432** | 0.056 | 0.050 | -0.115 | 0.260 | 0.262 | 0.044 |
| 35-methylhexatricont-10-ene | C_37br_ | 0.076 | 0.140 | 0.075 | 0.044 | -0.055 | 0.130 | 0.128 | 0.044 |
| 9,27-heptatricontadiene | C_36.5_ | 0.142 | 0.183 | 0.052 | 0.122 | -0.135 | 0.140 | 0.169 | 0.105 |
| 8,28-heptatricontadiene | C_36.6_ | 0.075 | 0.192 | 0.031 | 0.101 | -0.215 | 0.226 | 0.271 | -0.022 |
| 14-, 16-, and 12-hexatricontene | C_36.7_ | -0.014 | **0.225** | -0.002 | -0.061 | -0.036 | 0.056 | 0.141 | -0.006 |
| Unknown alkene | C_38_ | 0.132 | **0.615** | 0.187 | 0.058 | 0.168 | 0.211 | 0.242 | 0.065 |
| Unknown alkene | C_39_ | **0.205** | 0.273 | 0.265 | 0.167 | -0.301 | 0.143 | 0.167 | -0.017 |
| Unknown alkene | C_40_ | -0.107 | 0.184 | 0.012 | 0.082 | -0.074 | 0.102 | 0.151 | 0.006 |

^1^ Equivalent chain length for each hydrocarbon component.
