# Supplementary material for: Effects of temperature on transcriptome and cuticular hydrocarbon expression in ecologically differentiated populations of desert Drosophila
Source: Ecol Evol. 2016 Dec 20;7(2):619–37. doi: 10.1002/ece3.2653 (PMC5243788; doi:10.1002/ece3.2653)
Supplement: Supplementary file 10 [file ECE3-7-619-s010.docx]

Supplementary Table 9. Gene ontology and enrichment for the effects of Cactus X Temperature interactions on gene expression differences in female *D. mojavensis* in this study. All functional clustering was based on genes with FDR P < 0.01 for each treatment effect.

| Comparison | | No. Genes  (No. Annotated) | GOTerm | Enrich score |
| --- | --- | --- | --- | --- |
| 1. Cactus X Temperature | | 4837 (3536)^1^ | 1. peptidase activity, serine hydrolase  2. peptidase inhibitor activity  3. tetrapyrrole binding, heme binding  4. lysosome, glycan degradation  5. response to heat  6. aminoglycan metabolic process  7. exopeptidase activity  8. extracellular PGRP | 12.8****  9.7****  9.7****  3.2***  2.9**  2.9**  2.2**  1.5* |
| Agria - 15° > Agria - 25°  Agria - 15° < Agria - 25° | | 93 (76)  41 (26) | 1. chorion  2. peptidase inhibitor activity  3. nucleotide biosynthesis  *none* | 4.7***  3.0**  1.3*  - |
| Agria - 15° > Agria - 35°  Agria - 15° < Agria - 35° | 279 (215)  147 (120) | 1. endopeptidase inhibitor activity  2. tetrapyrrole binding, P-450 gene activity  3. pheromone, odorant binding  4. aminoglycan metabolic process  1. Heat shock protein Hsp20 | 6.2****  1.7*  1.4 *  1.3*  3.7*** |  |
| Agria - 25° > Agria - 35°  Agria - 25° < Agria - 35° | 116 (89)  90 (70) | 1. tetrapyrrole binding, P-450 gene activity  2. lipase, hydrolase  1. Heat shock protein Hsp20 | 1.5*  1.4*  4.2*** |  |
| Agria - 15° > Organ pipe - 25°  Agria - 15° < Organ pipe - 25° | 459 (333)  28 (13) | 1. endopeptidase inhibitor activity  2. peptidase activity, serine hydrolase  3. peptidoglycan recognition protein, PGRP-S  4. glycoside hydrolase  5. CHK kinase-like  6. amino acid transport and metabolism  7. metallopeptidase activity  1. vitelline membrane, chitin metabolism | 5.1****  2.9**  2.4**  2.4**  2.2**  2.1**  1.8*  - |  |
| Agria - 15° > Organ pipe - 35°  Agria - 15° < Organ pipe - 35° | | 683 (531)  76 (59) | 1. endopeptidase inhibitor activity  2. tetrapyrrole binding, P-450 gene activity  3. proteinase inhibitor I1, Kazal  4. peptidase activity, serine hydrolase  5. juvenile hormone binding protein  6. CHK kinase-like  7. exopeptidase activity  1. heat shock protein Hsp20 | 11.0****  4.0***  3.9***  3.1***  2.9**  2.0*  1.6*  4.7**** |
| Agria - 25° > Organ pipe - 15°  Agria - 25° < Organ pipe - 15° | | 72 (51)  66 (52) | 1. tetrapyrrole binding, P-450 gene activity  1. chorion | -  5.2**** |
| Agria - 25° > Organ pipe - 25°  Agria - 25° < Organ pipe - 25° | | 80 (60)  0 | 1. peptidase activity, serine hydrolase | 2.4** |
| Agria - 25° > Organ pipe - 35°  Agria - 25° < Organ pipe - 35° | | 466 (364)  37 (31) | 1. peptidase S1A, chymotrypsin  2. tetrapyrrole binding, P-450 gene activity  3. pheromone/odorant binding protein  4. CHK kinase-like  5. sugar transmembrane transporter  6. endopeptidase inhibitor activity  7. juvenile hormone binding protein  1. heat shock protein Hsp20  2. heat shock protein Hsp70 | 4.6****  4.3****  3.1***  2.8**  2.4**  2.3**  2.0**  5.4****  1.1 |
| Agria - 35° > Organ pipe - 15°  Agria - 35° < Organ pipe - 15° | | 259 (211)  159 (123) | 1. heat shock protein Hsp20  2. endopeptidase activity  3. $\alpha$ amylase domain, glycosyl hydrolase  4. metalloendopeptidase activity  5. tetrapyrrole binding, P-450 gene activity  1. endopeptidase inhibitor activity | 4.3****  3.3***  3.0***  2.2**  2.1**  4.2**** |
| Agria - 35° > Organ pipe - 25°  Agria - 35° < Organ pipe - 25° | | 364 (269)  25 (15) | 1. endopeptidase activity  2. Heat shock protein Hsp20  3. lysosome, glycan degradation  4. Peptidoglycan recognition protein, PGRP-S  5. serine protease  6. metalloendopeptidase activity  7. $\alpha$ amylase domain, glycosyl hydrolase  *none* | 6.6****  5.3****  3.7***  2.5**  2.3**  1.9*  1.6* |
| Organ pipe - 15° > Organ pipe - 25°  Organ pipe - 15° < Organ pipe - 25° | | 508 (392)  23 (13) | 1. endopeptidase inhibitor activity  2. tetrapyrrole binding, P-450 gene activity  3. juvenile hormone binding protein  4. proteinase inhibitor I1, Kazal  5. metallopeptidase activity  1. vitelline membrane | 7.7****  2.8**  2.4**  2.2**  2.0**  - |
| Organ pipe - 15° > Organ pipe - 35°  Organ pipe - 15° < Organ pipe - 35° | | 347 (267)  81 (67) | 1. endopeptidase inhibitor activity  2. tetrapyrrole binding, P-450 gene activity  3. proteinase inhibitor I1, Kazal  4. endopeptidase activity  5. Juvenile hormone binding protein  6. metalloendopeptidase activity  1. Heat shock protein Hsp20  2. oxidation reduction, heme binding | 8.2****  2.3**  2.3**  2.1**  1.6*  1.5*  4.7****  1.6* |

* P < 0.05, ** P < 0.01, *** P < 0.001, **** P < 0.0001

^1^ There were 1368 unique genes that exceeded 1.5 X fold change
