# Supplementary material for: Effects of temperature on transcriptome and cuticular hydrocarbon expression in ecologically differentiated populations of desert Drosophila
Source: Ecol Evol. 2016 Dec 20;7(2):619–37. doi: 10.1002/ece3.2653 (PMC5243788; doi:10.1002/ece3.2653)
Supplement: Supplementary file 12 [file ECE3-7-619-s012.docx]

Supplementary Table 11. Gene ontology and enrichment for the effects of Region X Temperature interactions on gene expression differences in female *D. mojavensis* in this study. All functional clustering was based on genes with FDR P < 0.01 for each treatment effect.

| Comparison | | No. Genes  (No. Annotated) | GOTerm | Enrich score |
| --- | --- | --- | --- | --- |
| 1. Region X Temperature | | 1124 (789) | 1. tetrapyrrole binding, heme binding  2. peptidase activity, serine hydrolase  3. endopeptidase inhibitor activity  4. aminoglycan metabolic process  5. response to heat  6. vitamin B6 binding | 7.6****  6.2****  5.7****  2.6**  2.3**  1.6* |
| Baja - 15° > Baja - 25°  Baja - 15° < Baja - 25° | | 302 (235)  121 (88) | 1. endopeptidase inhibitor activity  2. amino acid transport and metabolism  3. aminoglycan metabolic process, chitin  4. P-450 gene activity  *none* | 2.7**  2.0**  1.7*  1.4* |
| Baja - 15° > Baja - 35°  Baja - 15° < Baja - 35° | 1168 (932)  563 (417) | 1. endopeptidase inhibitor activity  2. tetrapyrrole binding, P-450 gene activity  3. CHK kinase-like  4. cellular retinaldehyde binding/alpha- tocopherol transport  5. proteinase inhibitor I2, Kazal type  6. juvenile hormone binding protein  7. hexose metabolic process  8. glutathione S-transferase  1. heat shock protein Hsp20  2. heat shock protein Hsp70  3. zinc finger, C2H2-type  4. zinc ion binding  5. lipid catabolism | 6.5****  4.3****  2.9**  2.7**  2.4**  2.2**  2.2**  1.6*  3.2***  2.7**  2.2**  1.9*  1.6* |  |
| Baja - 25° > Baja - 35°  Baja - 25° < Baja - 35° | 824 (676)  385 (276) | 1. tetrapyrrole binding, P-450 gene activity  2. ribosome  3. NAD(P)-binding domain, glucose dehydrogenase  4. endopeptidase inhibitor activity  5. juvenile hormone binding protein  6. CHK kinase-like  1. heat shock protein Hsp20  2. heat shock protein Hsp70 | 4.2****  3.9***  3.2***  2.5**  2.4**  2.1**  5.2****  2.9*** |  |
| Baja - 15° > Mainland - 15°  Baja - 15° < Mainland - 15° | 206 (134)  157 (86) | 1. acyltransferase 3  2. exopeptidase activity  3. NAD(P)-binding domain, glucose dehydrogenase  1. tetrapyrrole binding, P-450 gene activity  2. glutathione S-transferase | 2.3**  1.6*  1.4**  2.0**  1.5* |  |
| Baja - 15° > Mainland - 25°  Baja - 15° < Mainland - 25° | | 801 (556)  450 (271) | 1. peptidase M13  2. proteinase inhibitor I2, Kunitz  3. transmembrane helix  4. peptidase S1A, chymotrypsin-type  5. metalloprotease  6. C-type lectin fold  7. amino acid biosynthesis  8. secreted lipase  9. serpins  1. glycosyltransferase | 3.0***  2.4**  2.0**  1.8*  1.5*  1.5*  1.5*  1.5*  1.5*  2.6** |
| Baja - 15° > Mainland - 35°  Baja - 15° < Mainland - 35° | | 1199 (875)  751 (539) | 1. cytochrome P-450 gene activity  2. proteinase inhibitor I2, Kunitz  3. transmembrane helix  4. peptidase M13  5. cellular retinaldehyde binding/alpha- tocopherol transport  6. acyltransferase 3  7. secreted lipase  1. heat shock protein Hsp20  2. glutathione S-transferase  3. glycosyltransferase  4. protein folding, chaperone DnaJ  5. heat shock protein 70 | 4.2****  3.3***  2.6**  2.4**  2.4**  2.3**  1.6*  3.9***  2.4**  2.3**  1.9*  1.9* |
| Baja - 25° > Mainland - 15°  Baja - 25° < Mainland - 15° | | 466 (281)  416 (310) | 1. metalloendopeptidase activity  2. juvenile hormone binding protein  1. cytochrome P-450 gene activity  2. endopeptidase inhibitor activity  3. CHK kinase-like  4. glutathione S-transferase | 2.0**  1.6*  3.2***  2.9**  2.2**  1.9* |
| Baja - 25° > Mainland - 25°  Baja - 25° < Mainland - 25° | | 257 (174)  179 (110) | 1. peptidase activity, serine hydrolase  2. NAD(P)-binding domain  1. glutathione S-transferase | 2.9**  1.5*  2.3** |
| Baja - 25° > Mainland - 35°  Baja - 25° < Mainland - 35° | | 820 (607)  558 (400) | 1. heme, cytochrome P-450  2. secreted lipase  3. ribosome  4. transmembrane helix  5. glycosyltransferase  1. heat shock protein Hsp20  2. glycosyltransferase  3. glutathione S-transferase  4. heat shock protein 70 family | 3.8***  2.5**  2.2**  2.1**  1.5*  4.6****  2.5**  2.1**  2.0** |
| Baja - 35° > Mainland - 15°  Baja - 35° < Mainland - 15° | | 806 (557)  1299 (1051) | 1. heat shock protein Hsp20  2. metalloendopeptidase activity  3. nucleotide catabolism  4. zinc ion binding  5. heat shock protein 70  1. endopeptidase inhibitor activity  2. tetrapyrrole binding, P-450 gene activity  3. glutathione S-transferase  4. CHK kinase-like  5. juvenile hormone binding protein  6. proteinase inhibitor I2, Kazal type  7. pentose shunt  8. cellular retinaldehyde binding  9. fatty acid desaturase | 2.8**  2.0**  1.6*  1.6*  1.6*  5.1****  3.7***  3.7***  3.7***  2.6**  2.1**  1.8*  1.8*  1.6* |
| Baja - 35° > Mainland - 25°  Baja - 35° < Mainland - 25° | | 746 (494)  844 (654) | 1. heat shock protein Hsp20  2. heat shock protein Hsp70  3. peptidase activity, serine hydrolase  4. metalloendopeptidase activity  1. tetrapyrrole binding, P-450 gene activity  2. juvenile hormone binding protein  3. NAD(P)-binding domain  4. endopeptidase inhibitor activity  5. ribosome/translation | 4.0****  3.1***  2.4**  2.2**  3.7***  3.1***  2.3**  2.3**  1.9* |
| Baja - 35° > Mainland - 35°  Baja - 35° < Mainland - 35° | | 229 (145)  486 (182) | 1. peptidase M13  2. aspartic peptidase  3. metallopeptidase  1. heme, Cytochrome P-450  2. glutathione S-transferase  3. glycosyltransferase | 2.8**  2.4**  1.8*  5.6****  5.2****  5.1**** |
| Mainland - 15° > Mainland - 25°  Mainland - 15° < Mainland - 25° | | 353 (266)  105 (76) | 1. proteinase inhibitor I2, Kunitz  2. amino acid biosynthesis  3. amino acid transport  1. transmembrane helix | 3.6****  2.2**  1.6*  2.1** |
| Mainland - 15° > Mainland - 35°  Mainland - 15° < Mainland - 35° | | 812 (641)  81 (67) | 1. proteinase inhibitor I2, Kunitz  2. P-450 gene activity  3. Kazal domain  1. heat shock protein Hsp20  2. glycosyltransferase  3. heat shock protein 70 family | 3.3***  3.0***  2.3**  3.4***  2.2**  1.7* |
| Mainland - 25° > Mainland - 35°  Mainland - 25° < Mainland - 35° | | 330 (270)  415 (308) | 1. cytochrome P-450  2. CRAL-TRIO domain  3. proteinase inhibitor I29  4. fatty acid metabolism  1. heat shock protein Hsp20  2. heat shock protein 70 family  3. glycoside hydrolase, superfamily | 6.1****  1.8*  1.8*  1.5*  5.2****  2.3**  1.5* |

* P < 0.05, ** P < 0.01, *** P < 0.001, **** P < 0.0001
