# Supplementary material for: Effects of temperature on transcriptome and cuticular hydrocarbon expression in ecologically differentiated populations of desert Drosophila
Source: Ecol Evol. 2016 Dec 20;7(2):619–37. doi: 10.1002/ece3.2653 (PMC5243788; doi:10.1002/ece3.2653)
Supplement: Supplementary file 14 [file ECE3-7-619-s014.docx]

Supplementary Table 13. Gene ontology and enrichment for the effects of Region X Cactus interaction on gene expression differences in female *D. mojavensis* in this study. All functional clustering was based on genes with FDR P < 0.01 for each treatment effect.

| Comparison | | No. Genes  (No. Annotated) | GOTerm | Enrich score |
| --- | --- | --- | --- | --- |
| 1. Region X Cactus | | 3999 (2711) | 1. peptidase, proteolysis  2. tetrapyrrole binding, P-450 gene activity  3. peptidoglycan recognition proteins, microbial immunity | 12.6****  4.5****  1.7* |
| BC^1^ -Agria > BC Organ pipe  BC-Agria < BC Organ pipe | | 1127 (784)  192 (121) | 1. membrane  2. Immunoglobulin domain  3. glycosylation, signal peptide  4. neuron development  5. epithelial cell migration, open tracheal system development  6. ion transport  7. imaginal disc development  8. amino acid transport  9. leucine-rich repeat, protein binding  10. salivary gland development  1. histone, nucleosome assembly  2. cuticle protein | 15.7****  5.8****  5.6****  5.3****  5.0****  4.9***  4.5***  4.1***  3.7**  3.6**  3.0**  14.7****  1.6** |
| BC-Agria > Mainland-Agria  BC-Agria < Mainland-Agria | 447 (273)  347 (203) | 1. membrane  2. amino acid transport  3. ion transport  4. leucine-rich repeat, protein binding  5. metalloendopeptidase activity  6. signal peptide  7. Toll signaling pathway  1. Glutathione S-transferase  2. tetrapyrrole binding, P-450 gene activity  3. CHK kinase-like  4. endopeptidase inhibitor activity | 2.7**  2.4**  2.1**  1.7*  1.6*  1.5*  1.5*  4.4***  2.8**  1.4*  1.4* |  |
| BC-Organ pipe > Mainland-Agria  BC-Organ pipe < Mainland-Agria | 290 (170)  750 (584) | 1. nucleosome assembly  1. tetrapyrrole binding, P-450 gene activity, secondary metabolites biosynthesis  2. CHK kinase-like  3. Glutathione S-transferase  4. juvenile hormone binding protein  5. Glucose/ribitol dehydrogenase  6. transmembrane function  7. leucine-rich repeat, protein binding  8. pyridoxal phosphate binding  9. serine-type endopeptidase activity  10. sugar transporter | 4.0***  6.9****  5.3****  5.3****  3.7***  3.5***  2.8**  2.6**  2.3**  2.1**  2.0** |  |
| BC-Agria > Mainland-Organ pipe  BC-Agria < Mainland-Organ pipe | 1918 (1380)  951 (619) | 1. ATP synthesis  2. immunoglobulin-like  3. transmembrane  4. ion transport  5. neurotransmitter binding, synapse  6. signal peptide  7. neuron development  8. extracellular matrix  9. Fibronectin, type III  10. glycoprotein  1. DNA repair  2. DNA replication  3. mitosis  4. WD40 repeat 2  5. ATP binding  6. tRNA aminoacylation  7. zinc finger binding | 11.0****  10.1****  8.9****  6.9****  5.2****  4.2****  3.9***  3.4**  3.2**  3.1**  5.3****  3.6***  2.8**  2.5**  2.4**  2.1**  1.5* |  |
| BC-Organ pipe > Mainland-Organ pipe  BC-Organ pipe < Mainland-Organ pipe | | 314 (200)  317 (227) | 1. endopeptidase activity  2. metalloendopeptidase activity  1. P-450 gene activity, secondary metabolites biosynthesis  2. Glutathione S-transferase  3. tRNA processing  4. leucine-rich repeat, protein binding | 1.8*  1.4*  3.0***  3.0***  2.6**  1.4* |
| Mainland-agria > Mainland Organ pipe  Mainland-agria < Mainland Organ pipe | | 476 (396)  31 (25) | 1. peptidase, proteolysis, serine protease  2. oxidation reduction  3. mitochondrial oxidative phosphorylation  4. pheromone/odorant binding protein  5. CHK kinase-like  6. electron transport, mitochondria  7. exopeptidase activity  1. DNA replication, transcription, recombination | 5.7****  4.4****  3.8***  3.5***  3.5***  2.4**  2.2**  - |

* P < 0.05, ** P < 0.01, *** P < 0.001, **** P < 0.0001

^1^ Baja California
